# N‐terminomics profiling of naïve and inflamed murine colon reveals proteolytic signatures of legumain

**DOI:** 10.1002/jcp.31466

**Published:** 2024-10-11

**Authors:** Alexander R. Ziegler, Bethany M. Anderson, Rocco Latorre, Rachel M. McQuade, Antoine Dufour, Brian L. Schmidt, Nigel W. Bunnett, Nichollas E. Scott, Laura E. Edgington‐Mitchell

**Affiliations:** ^1^ Department of Biochemistry and Pharmacology, Bio21 Molecular Science and Biotechnology Institute The University of Melbourne Parkville Victoria Australia; ^2^ Department of Molecular Pathobiology New York University College of Dentistry New York New York USA; ^3^ Department of Anatomy and Physiology The University of Melbourne Parkville Victoria Australia; ^4^ Department of Physiology and Pharmacology University of Calgary Calgary Alberta Canada; ^5^ Department of Oral and Maxillofacial Surgery New York University College of Dentistry, Bluestone Center for Clinical Research New York New York USA; ^6^ Department of Microbiology and Immunology, Peter Doherty Institute The University of Melbourne Parkville Victoria Australia

**Keywords:** activity‐based probes, colitis, functional imaging, inflammatory bowel disease, legumain, N‐terminomics

## Abstract

Legumain is a cysteine protease broadly associated with inflammation. It has been reported to cleave and activate protease‐activated receptor 2 to provoke pain associated with oral cancer. Outside of gastric and colon cancer, little has been reported on the roles of legumain within the gastrointestinal tract. Using a legumain‐selective activity‐based probe, LE28, we report that legumain is activated within colonocytes and macrophages of the murine colon, and that it is upregulated in models of acute experimental colitis. We demonstrated that loss of legumain activity in colonocytes, either through pharmacological inhibition or gene deletion, had no impact on epithelial permeability in vitro. Moreover, legumain inhibition or deletion had no obvious impacts on symptoms or histological features associated with dextran sulfate sodium‐induced colitis, suggesting its proteolytic activity is dispensable for colitis initiation. To gain insight into potential functions of legumain within the colon, we performed field asymmetric waveform ion mobility spectrometry‐facilitated quantitative proteomics and N‐terminomics analyses on naïve and inflamed colon tissue from wild‐type and legumain‐deficient mice. We identified 16 altered cleavage sites with an asparaginyl endopeptidase signature that may be direct substrates of legumain and a further 16 cleavage sites that may be indirectly mediated by legumain. We also analyzed changes in protein abundance and proteolytic events broadly associated with colitis in the gut, which permitted comparison to recent analyses on mucosal biopsies from patients with inflammatory bowel disease. Collectively, these results shed light on potential functions of legumain and highlight its potential roles in the transition from inflammation to colorectal cancer.

## 1 INTRODUCTION

Protease‐activated receptor 2 (PAR_2_) is a G protein‐coupled receptor that is uniquely activated by proteolytic cleavage (Peach et al., 2023). A critical regulator of gastrointestinal function, its signaling influences epithelial permeability and proliferation, inflammation, and neuronal sensitivity in the gut (Cenac et al., 2007; Gecse et al., 2008; Hyun et al., 2008; Jimenez‐Vargas et al., 2018; Latorre, Hegron, et al., 2022; Lohman et al., 2012; Roka et al., 2007). Mice lacking PAR_2_ were protected from symptoms of experimental colitis induced by 2,4,6‐trinitrobenzene sulfonic acid (TNBS), with reduced weight loss, myeloperoxidase (MPO) activity, colon thickening, and microscopic damage compared to wild‐type mice (Hyun et al., 2008). A small molecule PAR_2_ antagonist, GB88, abrogated colon shortening, ulceration, edema, and bowel obstruction in this model, suggesting therapeutic value of targeting the PAR_2_ signaling pathway (Lohman et al., 2012). Various lines of evidence also suggest that PAR_2_ is implicated in the pathogenesis of colorectal cancer (Lv et al., 2023).

Legumain is a cysteine protease that has recently been identified as a novel PAR_2_‐activating protease (Tu et al., 2021). It cleaves the N terminus of PAR_2_ at N^30^ to provoke neuronal excitation and nocifensive behaviors in multiple mouse models. Legumain‐deficient mice were protected from oral cancer‐induced pain, and a legumain inhibitor exhibited analgesic effects. Legumain activity is associated with numerous inflammatory diseases, including pancreatitis, atherosclerosis, neuroinflammation, and cancer (Edgington‐Mitchell et al., 2016; Lei et al., 2021; Lunde et al., 2017; Z. H. Wang et al., 2017; Z. H. Wang et al., 2021; Wu et al., 2020; Xia et al., 2020). Given its ability to activate PAR_2_, and that PAR_2_ activation clearly underlies colitis pathophysiology, we hypothesized that legumain may also play key roles in this setting. Outside the context of gastric and colorectal cancers, where its high expression correlates with worse prognoses (Cui et al., 2016; Cui et al., 2017; Guo et al., 2013; Haugen et al., 2013; Haugen et al., 2015; Kovalyova et al., 2022; N. Li et al., 2013; Murthy et al., 2005; H. Wang et al., 2020; Xu, Liu, et al., 2023), studies of gastrointestinal legumain activity have not been previously reported.

Herein, we applied activity‐based probes to demonstrate that legumain is active in mouse colon tissue, where its predominant sources are macrophages and colonocytes. Legumain is significantly upregulated in models of acute experimental colitis. Loss of legumain activity, either through pharmacological inhibition or genetic deletion, was not protective against symptoms of colitis, suggesting it is dispensable for initiation of pathogenesis. To gain insight into potential functions of legumain in the colon, we used a systematic and unbiased proteomics approach to examine its influence on total protein abundance and on proteolysis. We further extended this analysis to broadly profile protease cleavage events that are altered in the landscape of acute colitis. Collectively, our results inform future mechanistic studies on legumain in the gut, especially its implications in cancer development, and allow comparison of mouse models of acute colitis with human mucosal biopsies.

## 2 METHODS

### 2.1 Mice

Studies involving animals were approved by and carried out in accordance with the guidelines of the Animal Ethics Committee at Monash University or the New York University Institutional Animal Care and Use Committee. Wild‐type C57Bl/6 J mice were purchased from the Monash University in‐house colony or purchased from JAX (#000664). Legumain‐deficient mice (Matthews et al., 2010) were kindly provided by Thomas Reinheckel and bred in the New York University animal facility. All mice were maintained with free access to food and water under temperature and light controlled conditions.

### 2.2 Dextran sulfate sodium (DSS)‐induced colitis

Acute colitis was induced in 8–10‐week‐old male mice by administering 3% DSS (MP Biomedicals 36,000–50,000 Da) in the drinking water for 6 days. Control (naïve) mice received normal drinking water. Body weights and symptoms were monitored daily. Body weight loss was scored (0–none; 1–1%–5%; 2–5%–10%). Fecal pellets were scored using scales for consistency (0–dry; 1–moist but firm; 2–soft but still formed; 3–unformed but bulky; 4–liquid) and blood (0–no blood; 1–subtle blood or darkening of the stool; 2–red streaks in stool; 4–obvious rectal bleeding). Disease activity index (DAI) was calculated by adding scores for weight consistency and blood on each day. For the legumain inhibitor trial, LI‐1 (Edgington‐Mitchell et al., 2016; Lee & Bogyo, 2012) (25 mg/kg in 100 µL PBS containing 20% DMSO) or vehicle control was administered daily by intraperitoneal injection. Unless otherwise indicated, colons were harvested on Day 6. Colons were flushed with phosphate buffered saline (PBS). Luminal fluid was collected, centrifuged to clear solids, and frozen. Colon tissue was divided and frozen for gel analysis and MPO assay or fixed for microscopy or histology. Where indicated, mucosa and muscle tissue were separated before freezing.

### 2.3 TNBS‐induced colitis

Male mice (8–10 weeks old male mice) were sedated using isofluorane. Using a PE10 catheter inserted 4 cm into the rectum, mice were first infused with a saline enema, followed by 100 µL of 2,4,6‐trinitrobenzene sulfonic acid (TNBS; 2 mg/mouse, 50% ethanol/saline, 100 µL) or vehicle (50% ethanol/saline, 100 µL). On Day 3, colons were harvested and processed as above.

### 2.4 Mechanical allodynia

Mice were acclimatized to the room, apparatus, and investigator for 2 h per day for 2 days before the study. To assess colonic nociception, the abdominal area was divided into 9 equal quadrants; von Frey filaments of increasing force (0.07–2 g) were applied to the central quadrant, which corresponds to the area of the colon (Latorre, Hegron, et al., 2022). Responses to von Frey filament were arching of the back, jumping, and raising the rear legs. Responses were measured on Day 0, before the beginning of the DSS colitis treatment, and at end point on Day 6. Results are expressed as a mechanical threshold in grams.

### 2.5 Spontaneous nonevoked pain behavior

To measure nonevoked pain behavior, we used a behavioral spectrometer (Behavior Sequencer by Behavioral Instruments, NJ, and BiObserve by DE) (Latorre, Ramírez‐Garcia, et al., 2022). This instrument quantifies the locomotor, exploratory, and grooming behavior of mice, eliminating operator bias. The mice were placed individually in the center of the behavioral spectrometer and their behavior was recorded, tracked, evaluated, and analyzed for 20 min using a computerized video tracking system (Viewer3 by BiObserve, DE). We recorded and analyzed the total distance traveled in the open field, average velocity of locomotion, wall distance, ambulation, and grooming.

### 2.6 Ex vivo imaging of legumain activity

At endpoint, naïve, DSS‐ or TNBS‐treated mice were injected with the legumain activity‐based probe LE28 intravenously by tail vein (20 nmol in 100 µL 20% DMSO/PBS) (Edgington et al., 2013). After 6 h, colons were harvested, flushed with PBS, and imaged for Cy5 fluorescence using an IVIS Lumina XR III in vivo imaging system (Perkin Elmer). Proximal colons were fixed in 4% paraformaldehyde overnight at 4℃ followed by 30% sucrose. Tissues were then embedded in optimal cutting temperature (OCT) Compound (TissueTek) and frozen on dry ice. Ten‐micrometer sections were cut and air‐dried. Slides were submerged in cold acetone for 10 min and dried at room temperature for 10 min. OCT was dissolved with PBS and sections were blocked for 30 min in blocking buffer (PBS at pH 7.4, 3% normal horse serum, and 0.05% Triton X‐100). Sections were incubated with the indicated primary antibody in blocking buffer at 4℃ overnight followed by three washes with PBS: sheep anti‐mouse legumain (1:100, R&D Systems AF2058); rat anti‐mouse CD68 (1:500, clone FA‐11, BioLegend). Secondary antibodies (donkey anti‐sheep/rat‐AlexaFluor488, 1:500; Jackson ImmunoResearch) were added for 1 h at room temperature followed by DAPI staining (1 µg/mL) and three PBS washes. Sections were mounted with ProLong Gold (Life Technologies, Scoresby, Australia). Staining was visualized using a Leica SP8 inverted confocal microscope.

### 2.7 Analysis of activity‐based probe labeling by SDS‐PAGE

Mouse proximal colon tissues were lysed by sonication in PBS (for in vivo‐labeled samples) or citrate buffer (for in vitro‐labeled samples; 50 mM citrate, pH 5.5, 0.5% CHAPS, 0.1% Triton X‐100, 4 mM DTT). Lysates were cleared by centrifugation at 14,000 × *g* for 5 min at 4℃. Cleared luminal fluids were concentrated using a 3‐kDa cutoff column (Amicon). Fecal pellets were homogenized in PBS and centrifuged to clear solids. Total protein concentration was measured in colon lysate, luminal fluid and fecal supernatant using a BCA assay and diluted into PBS or citrate buffer (50 µg total protein in 20 µL). For in vivo‐labeled samples, 5× sample buffer was immediately added (50% glycerol, 250 mM Tris‐Cl, pH 6.8, 10% SDS, 0.04% bromophenol blue, 6.25% beta‐mercaptoethanol; 1× final). For in vitro‐labeled samples, LE28 (1 µM) was added from a 100× DMSO stock concentration and incubated at 37℃ for 30 min. The labeling reaction was quenched by addition of 5× sample buffer. Samples were boiled for 5 min and resolved on a 15% SDS‐PAGE gel. Gels were scanned for Cy5 fluorescence using a Typhoon 5 flatbed laser scanner (GE Healthcare). After transferring to nitrocellulose membranes, membranes were immunoblotted overnight with sheep anti‐legumain (1:1000; R&D; AF2058) or goat anti‐cathepsin L (1:1000, R&D, AF1515) followed by detection with donkey anti‐goat‐IR800 (1:10,000; LI‐COR; 9263‐2214). Membranes were scanned using a Typhoon 5.

### 2.8 Immunoprecipitation

LE28‐labeled samples were divided into input and pulldown, each containing 50 µg total protein. Pulldown samples were diluted in 500 µL immunoprecipitation (IP) buffer (PBS, pH 7.4, 0.5% Nonidet P 40 Substitute (Sigma), 1 mM EDTA) followed by 10 µL of sheep anti‐mouse legumain (R&D Systems AF2058). Protein A/G agarose beads (40 µL slurry; Santa Cruz Biotechnology) were washed in IP buffer, added to the sample, and rotated overnight at 4℃. Beads were washed four times in IP buffer and once in NaCl (0.9%) and resuspended in 20 µL 2× sample buffer. Input and pulldown were boiled for 5 min and resolved by fluorescent SDS‐PAGE as above.

### 2.9 Histology and immunohistochemistry

Colon tissues were fixed overnight at 4℃ in 4% paraformaldehyde in PBS before transferring to 70% ethanol for paraffin embedding. Tissues were sectioned, dewaxed and stained with hematoxylin and eosin according to standard protocols. Slides were scanned on a Mirax Digital Slide Scanner (Zeiss) by Australian Phenomics Network at The University of Melbourne. Slides were deidentified and four random regions from each colon were scored for crypt organization (0–5), immune cell infiltration (0–5) and goblet cell expression/cavitation (0–5) based on modified histomorphological evaluation criteria (Erben et al., 2014; McQuade et al., 2017). All images were analyzed blindly. For immunostaining, antigen retrieval was performed in 10 mM citrate buffer, pH 6.0 in the microwave for 15 min total without boiling. All slides were washed in PBS, followed by incubation in blocking buffer (3% normal horse serum in PBS‐Tween; PBST) for 30 min at room temperature. Tissues were incubated in anti‐mouse legumain antibody (R&D Systems AF2058) in a humidified chamber overnight at 4℃. Endogenous peroxidases were blocked using 1% hydrogen peroxide in PBS for 10 min. Following three washes in PBST, secondary antibody anti‐goat HRP (Invitrogen A15999; 1:500) was added at room temperature for 1 h. Tissues were washed twice with PBST and twice with PBS and stained with 3,3′‐Diaminobenzidine (DAB) (eBioscience DAB Advanced Chromogenic Kit) according to manufacturer's protocol. After 30 min, slides were placed in water to stop the reaction. Tissues were stained briefly with haematoxylin and then dehydrated by immersing three times in 100% ethanol and four times in xylene. Coverslips were mounted using Entellan (Merck) and slides were kept covered overnight to set. Slides were scanned as above.

### 2.10 MPO activity assay

Tissues from the middle region of the colon or spleen were assayed for MPO activity. Tissues were sonicated in buffer containing 50 mM potassium phosphate, pH 6.0, 0.5% hexadecyl trimethylammonium bromide (50 mg tissue per mL) and supernatants were cleared by centrifugation. In a 96 well plate, 7 µL sample was diluted in 193 µL substrate solution containing 50 mM potassium phosphate, pH 6.0, O‐dianisidine HCl (0.167 mg/mL) and 0.0005% H_2_O_2_. Absorbance at 460 nm was measured every 40 s for 25 min on a CLARIOstar Plus (BMG Labtech) and slopes were recorded.

### 2.11 Field asymmetric waveform ion mobility spectrometry (FAIMS)‐facilitated N‐terminomics

Quantitative proteomics and N‐terminomics analysis of colon tissue from wild‐type male C57BL/6 and *Lgmn* ^−/−^ mice (*n* = 4/group) were completed using a modification of our previously described method (Ziegler, Dufour, Scott, & Edgington‐Mitchell, 2024). Tissue lysis was facilitated by sonication in 4% SDS, 50 mM HEPES (pH 7.5, Sigma) containing Roche cOmplete, EDTA‐free protease inhibitor (Sigma) and subsequent boiling for 10 min. Lysates were cleared by centrifugation (21,000 × *g*, 5 min, 4℃) and total protein (100 µg) was diluted in 100 µL buffer according to BCA analysis.

Proteins were reduced with 20 mM DTT (80℃, 10 min, 500 rpm) and alkylated with 50 mM iodoacetamide (37℃, 30 min, 500 rpm) in the dark followed by quenching with 50 mM DTT (37℃, 20 min, 500 rpm). Conditioned paramagnetic SP3 beads (Sera‐Mag SpeedBeads 45152105050250 and 65152105050250, GE Healthcare) (Hughes et al., 2019) were added to samples (2 mg of SP3 beads, final protein: SP3 bead ratio of 1:20) with ethanol (80% final concentration) to facilitate protein aggregation and incubated for 20 min (25℃, 1000 rpm). Following three rounds of washing with 500 µL of 80% ethanol, peptides were resuspended in 90 µL 200 mM HEPES, pH 7.5. Primary amines were labeled at the protein level using TMTpro 16‐plex reagents (Thermo, #A44520) by adding 80 µg of each reagent diluted in 10 µL 100% ACN and incubating for 60 min (25℃, 1000 rpm, 4:5 TMTpro:protein) (Zecha et al., 2019). This was repeated once more before incubating with 3% hydroxylamine (2 µL) for a further 60 min (1000 rpm) to quench labeling. Excess reagents were removed from samples using SP3 clean up (1 mg SP3 beads; final protein: SP3 bead ratio of 1:30) and proteins were precipitated with ethanol (80% final concentration). Samples were gently shaken (25℃, 1000 rpm) for 20 min and then washed three times with 500 µL of 80% ethanol using a magnetic rack. SP3 beads were then resuspended in 100 μL of 200 mM HEPES (pH 7.5) and digested overnight at 37℃ with Solu‐trypsin (3 µg solu‐trypsin, Sigma, trypsin:protein ratio 1:33). The resulting peptide mixtures were collected using a magnetic rack, and pooled. The pooled sample was acidified with Buffer A* (0.1% trifluoroacetic acid, 2% acetonitrile) before desalting using a 50 mg Sep‐Pak Cartridge C18 Column (Waters, WAT054960) as previously described (Demir et al., 2017). Samples were dried using a speedvac and stored at −20℃ until analysis.

### 2.12 Online fractionation by high‐FAIMS and mass spectrometry (MS) analysis

Proteome samples were re‐suspended in Buffer A* and separated using a two‐column chromatography setup composed of a PepMap100 C_18_ 20‐mm by 75‐μm trap and a PepMap C_18_ 500‐mm by 75‐μm analytical column (Thermo Fisher Scientific) on a Dionex Ultimate 3000 UPLC (Thermo Fisher Scientific). Samples were concentrated onto the trap column at 5 μL/min for 5 min with Buffer A (0.1% formic acid, 2% DMSO) and then infused into an OrbiTrap Fusion Lumos mass spectrometer (Thermo Fisher Scientific) equipped with a FAIMS Pro interface at 300 nL/minute. For each sample/FAIMS fraction ~2 µg of peptide mixtures was separated using 125‐min analytical runs undertaken by altering the buffer composition from 3% Buffer B (0.1% formic acid, 77.9% acetonitrile, 2% DMSO) to 23% B over 95 min, then from 23% B to 40% B over 10 min, then from 40% B to 80% B over 5 min. The composition was held at 80% B for 5 min, and then dropped to 3% B over 0.1 min before being held at 3% B for another 9.9 min. For each sample, six individual LC‐MS runs were collected with the OrbiTrap Fusion Lumos mass spectrometer operated using different FAIMS compensational voltages (CV) of either −20, −30, −40, −50, −60 or −70. For each FAIMS fraction, data‐dependent acquisition was undertaken with a single Orbitrap MS scan (300–2000 m/z, a resolution of 60k with the automated gain control (AGC) set to a maximum of 400%) collected every 3 s followed by Orbitrap MS/MS HCD scans of precursors (Quad isolation window width of 1.6 m/z, stepped normalized collision energy of 35;38;45%, maximal injection time of 118 ms, a resolution of 60k and a AGC of 500%, lower mass cut off set at 120 m/z). The mass spectrometry proteomics data have been deposited to the Proteomexchange Consortium via the PRIDE (Perez‐Riverol et al., 2022) partner repository with the data set identifier PXD051470.

### 2.13 Bioinformatic analyses

Raw data files were processed and searched using MSFragger (Fragpipe v.21.0) (Kong et al., 2017) against the unreviewed murine proteome (*Mus musculus*, UniProt Accession: UP000000589, downloaded January 2024, 25,658 protein entries), supplemented with common contaminants, and a reverse decoy database (25,658 decoys: 50%). All six FAIMS fractions for a given sample were defined as a single biological replicate with individual FAIMS CVs defined as fractions and experiments searched all together to ensure a global false discovery rate of 1% (Schaab et al., 2012). Parameters were set to default unless otherwise described below. Identification and isobaric quantification were undertaken allowing for cysteine carbamidomethylation as a fixed modification (+57.0215 Da) as well as variable modifications of methionine oxidation (+15.9949 Da), N‐terminal acetylation (+42.0106 Da), N‐terminal cyclisation (−17.0265/−18.0106 Da), N‐terminal and lysine TMT‐labeling (+304.20715 Da), and N‐terminal lysine TMT‐labeling (+608.4143 Da). Cleavage specificity was set to “SEMI‐N_TERM” and “TrypsinR” (Arg‐C), allowing a maximum of two missed cleavages. Precursor and fragment mass tolerances of 20 ppm and isotopic error of 3 Da were also included. Protein and peptide‐level false discovery rates (FDR) were determined using Philosopher (v.5.1.0) with default settings (FDR threshold set at 1%). Isobaric TMT‐16 quantification parameters were left as default and performed with IonQuant (v.1.10.12) (Yu et al., 2021). Quantification level was set to two with a mass tolerance of 20 ppm and a virtual reference. The resulting outputs (MaxLFQ values) were further processed in Perseus (v.1.6.0.7) (Tyanova et al., 2016), where a log_2_ transformation was applied. Protein/peptides identified in a minimum of three of four biological replicates in at least one of the groups were selected and missing values imputed based on a downshifted normal distribution (σ‐width = 0.3, σ‐downshift = −1.8) for statistical analyses at the protein and N‐termini level. Due to legumain cleavage events being absent in *Lgmn* ^−/−^ samples, imputation was used to allow statistical analysis to guide the identification of cleavage events overrepresented within WT samples. Student's two‐sample *t*‐test was applied for statistical comparison between groups with a significance threshold set to log_2_(fold change) ±1 and −log_10_(p) = 1.3 (*p* = 0.05). Volcano plots and Venn diagrams were created using R (v.4.2.0).

Data were processed in WebPICS (Schilling & Overall, 2008) and TopFINDer (Fortelny et al., 2015) for generation of sequence logos using plogo (O'Shea et al., 2013). STRING‐db (v.12.0) was used for protein interaction and pathway analyses (https://stringdb.org) with medium confidence (0.400) and FDR stringency (5%).

### 2.14 Statistical analyses

All experiments were performed with at least 3 biological replicates. Data are reported as means ± SEM. Statistical significance was determined by the indicated test, and *p* values of less than 0.05 were considered significant.

## 3 RESULTS

### 3.1 Legumain activity is increased in the colon during acute experimental colitis

To study the activation of legumain in the setting of acute experimental colitis, we used the activity‐based probe LE28 (Edgington et al., 2013). LE28 is a fluorescently quenched probe that selectively and covalently binds to the active site cysteine of legumain in an activity‐dependent manner. Upon binding, a quenching group is released, leading to an increase in fluorescence of the Cy5 fluorophore. The fluorescence can be then detected as a surrogate readout of legumain activity, either by optical imaging of cells or tissues or by in‐gel analysis of probe‐labeled lysates.

**Figure 1:**
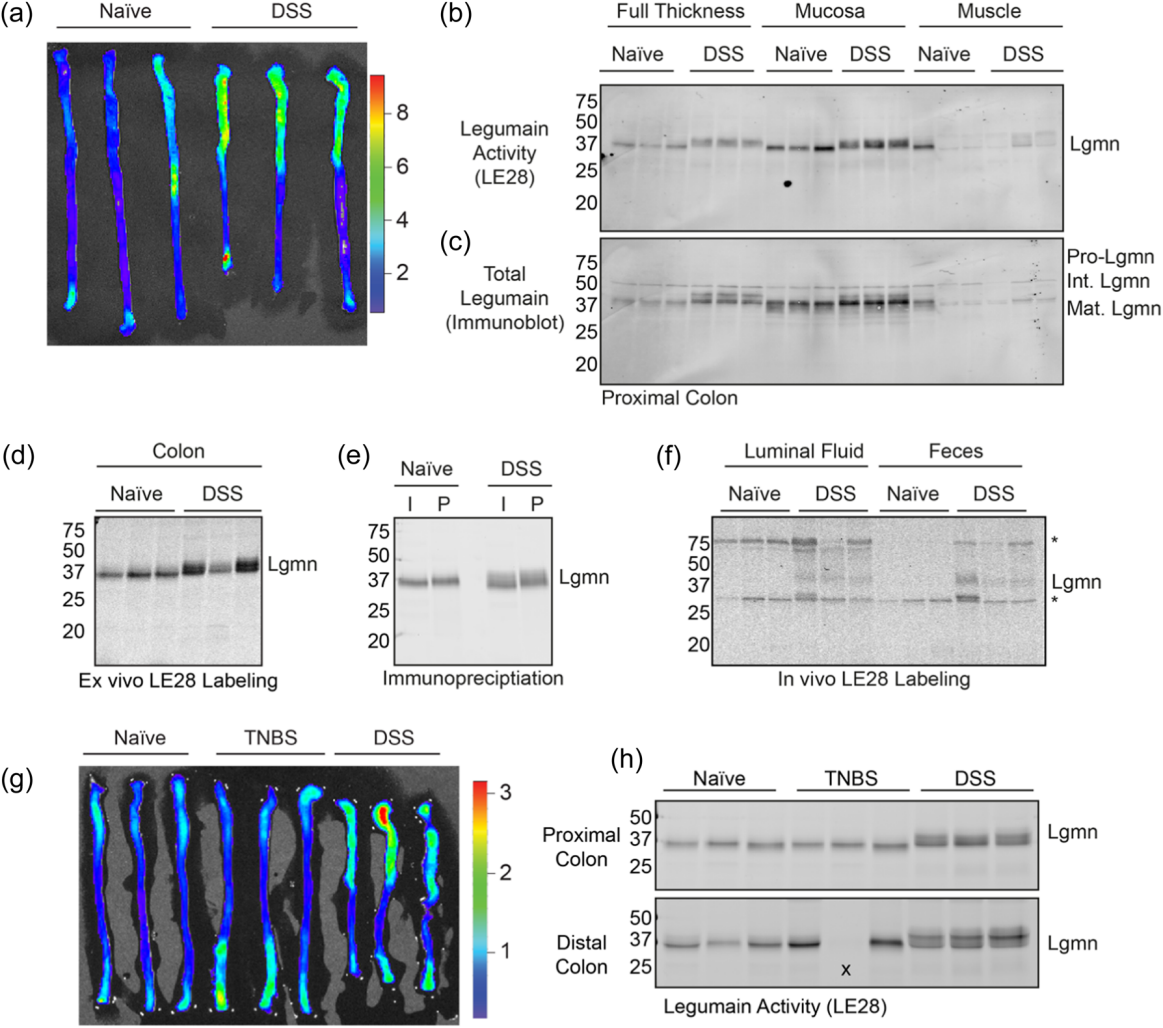
Mucosal legumain is increased during acute colitis. (a) Legumain activity in colons harvested from naïve mice or mice treated with DSS for 6 days, as shown by ex vivo imaging of LE28 fluorescence. Three biological replicates per group are shown. Color scale represents radiant efficiency/10^9^ (p/sec/cm^2^/sr)/(μW/cm^2^). (b) Legumain activity in proximal colon tissues shown in A, either full thickness, or dissected into mucosal and muscle layers, as shown by in‐gel fluorescence of LE28. (c) Immunoblot of gel shown in B with a legumain‐specific antibody. (d) Ex vivo labeling of lysates prepared from naïve and DSS‐treated colon tissue with LE28, as shown by in‐gel fluorescence. Three biological replicates per group are shown. (e) Immunoprecipitation of LE28‐labeled lysates prepared from full thickness colons in shown in A with a legumain‐specific antibody. I, input; P, pulldown. (f) In‐gel fluorescence showing LE28 labeling in luminal fluids and fecal samples from mice imaged in A. *Designates LE28‐labeled species of unknown identity. (g) LE28 fluorescence in naïve mice compared to those treated with TNBS for 3 days or DSS for 6 days. Three biological replicates are shown. (h) In‐gel fluorescence of the LE28‐labeled proximal and distal colons shown in H. Note the x designates a lost distal colon sample. Color scale represents radiant efficiency/10^8^ (p/sec/cm^2^/sr)/(μW/cm^2^).

Acute colitis was induced through administration of 3% DSS in drinking water. On day 6, LE28 was intravenously administered, and after 6 h, colons were removed, flushed, and imaged ex vivo for LE28 fluorescence. Fluorescence was increased in the proximal colons of DSS‐treated mice compared to naïve colons (Figure 1a). In‐gel fluorescence of full thickness colon lysates revealed increased legumain labeling by LE28 in DSS‐treated tissues compared to naïve (Figure 1b). When mucosal and muscle tissues were analyzed separately, it was evident that most of the legumain was confined to the mucosal layer (Figure 1b). In inflamed tissues, LE28‐labeled species were slightly larger than in naïve tissue, as we previously observed in the context of acute pancreatitis (Edgington‐Mitchell et al., 2016). This corresponded to an increase in total legumain levels detected by immunoblot and the appearance of a higher molecular weight form of legumain (Figure 1c). We also observed this phenomenon when legumain was labeled with LE28 ex vivo in colon lysates (Figure 1d). To verify the identity of the probe‐labeled species in both naïve and DSS‐treated colons, we immunoprecipitated LE28‐labeled lysates with a legumain‐specific antibody (Figure 1e). Indeed, the immunoprecipitated species mirrored the input samples, confirming specificity of LE28 for legumain in these samples.

We also analyzed luminal fluid and fecal samples collected from the mice after LE28 administration. In samples from naïve mice, labeling of 36 kDa legumain was virtually absent; however, it was clearly detected in DSS‐treated samples (Figure 1f). These results suggest that luminal secretion of legumain may increase during acute colitis. Additional proteins were labeled with LE28 (indicated by asterisks) in these samples, although the identity of these species is still unknown.

In addition to DSS, we also examined legumain in TNBS‐induced colitis. Increased legumain activity was observed in acute colitis induced by TNBS, although it was most active in the distal region as opposed to the proximal region, which is in line with locations affected by these models (Figure 1g–h). Together, these results suggest that legumain is activated in multiple models of acute experimental colitis.

### 3.2 Legumain is activated in macrophages and colonocytes

**Figure 2:**
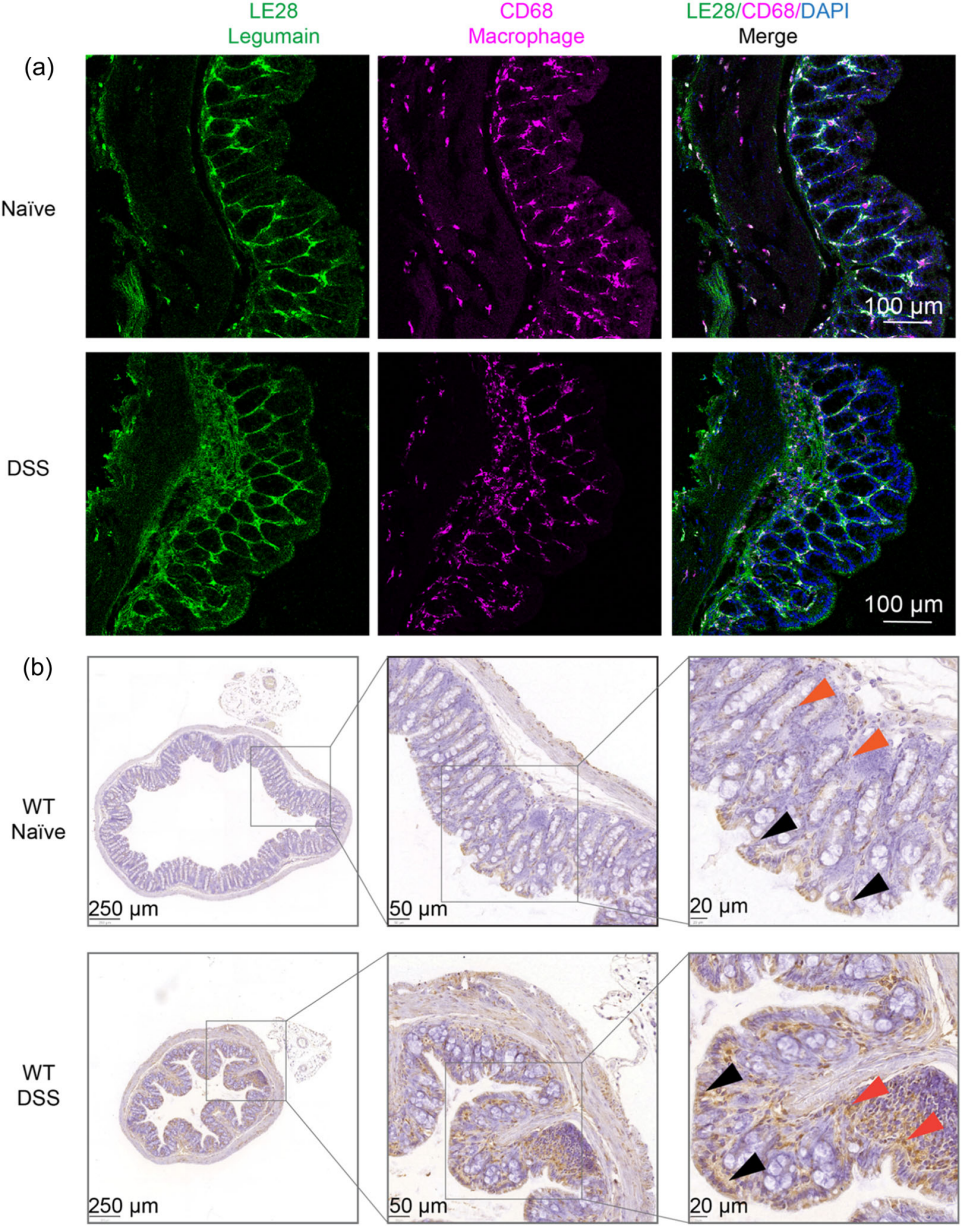
Legumain is present in macrophages and colonocytes of the colon. (a) LE28 fluorescence showing legumain activity (green), CD68 immunoreactivity (magenta), or the two merged with DAPI (blue) in colon tissue sections from naïve mice or those treated with DSS for 6 days. (b) Legumain immunoreactivity in colon tissue sections from naïve or DSS‐treated mice. Black arrowheads denote colonocytes while red show macrophages.

To determine the cellular source of legumain in the colon, we analyzed LE28‐labeled tissues by confocal microscopy. In both naïve and DSS‐treated colons, the majority of LE28 fluorescence was contained within CD68^+^ cells, suggesting that legumain in both mucosal and muscle layers is largely macrophage‐derived (Figure 2a). We also observed LE28 fluorescence in colonocytes. Immunohistochemistry of paraffin‐embedded colon tissues also revealed anti‐legumain reactivity in these cells as well as macrophage‐like cells throughout the mucosa (Figure 2b). Staining of tissues harvested from legumain‐deficient mice confirms the specificity of the anti‐legumain antibody used (Figure S1). Together, these results suggest that the major sources of legumain in the murine colon are macrophages and epithelial cells. This mirrors what was previously observed in normal human colon tissue adjacent to CRC (Haugen et al., 2015).

### 3.3 Colonocyte‐derived legumain may not regulate epithelial permeability

In light of our observation that legumain is present in colonocytes, we hypothesized that its activity may contribute to barrier function, either through activation of PAR_2_ or potentially through cleavage of other substrates that mediate epithelial adhesion. We first verified that legumain is present and active in the immortalized human colonocyte line Caco‐2 (Figure S2A,B). Legumain is also highly secreted from these cells, although under standard cell culture conditions, it remains in the inactive zymogen form (56 kDa). We demonstrated that that a legumain inhibitor SD‐134 (Jafari et al., 2017; Lee & Bogyo, 2012) could block its intracellular activity in wild‐type cells (Figure S2C). Using the CRISPR/Cas9 system (Figure S2D), we generated two legumain‐deficient Caco‐2 single‐cell clones (Figure S2E). In a permeability assay, we observed no differences in the flux of FITC‐dextran (3–5 kDa) between wild‐type and legumain‐deficient clones (Figure S2F–G). Likewise, treatment with SD‐134 had no clear effects on monolayer permeability. Thus, legumain may not regulate epithelial permeability, at least under these conditions in vitro.

### 3.4 Legumain inhibition does not attenuate acute colitis induced by DSS

**Figure 3:**
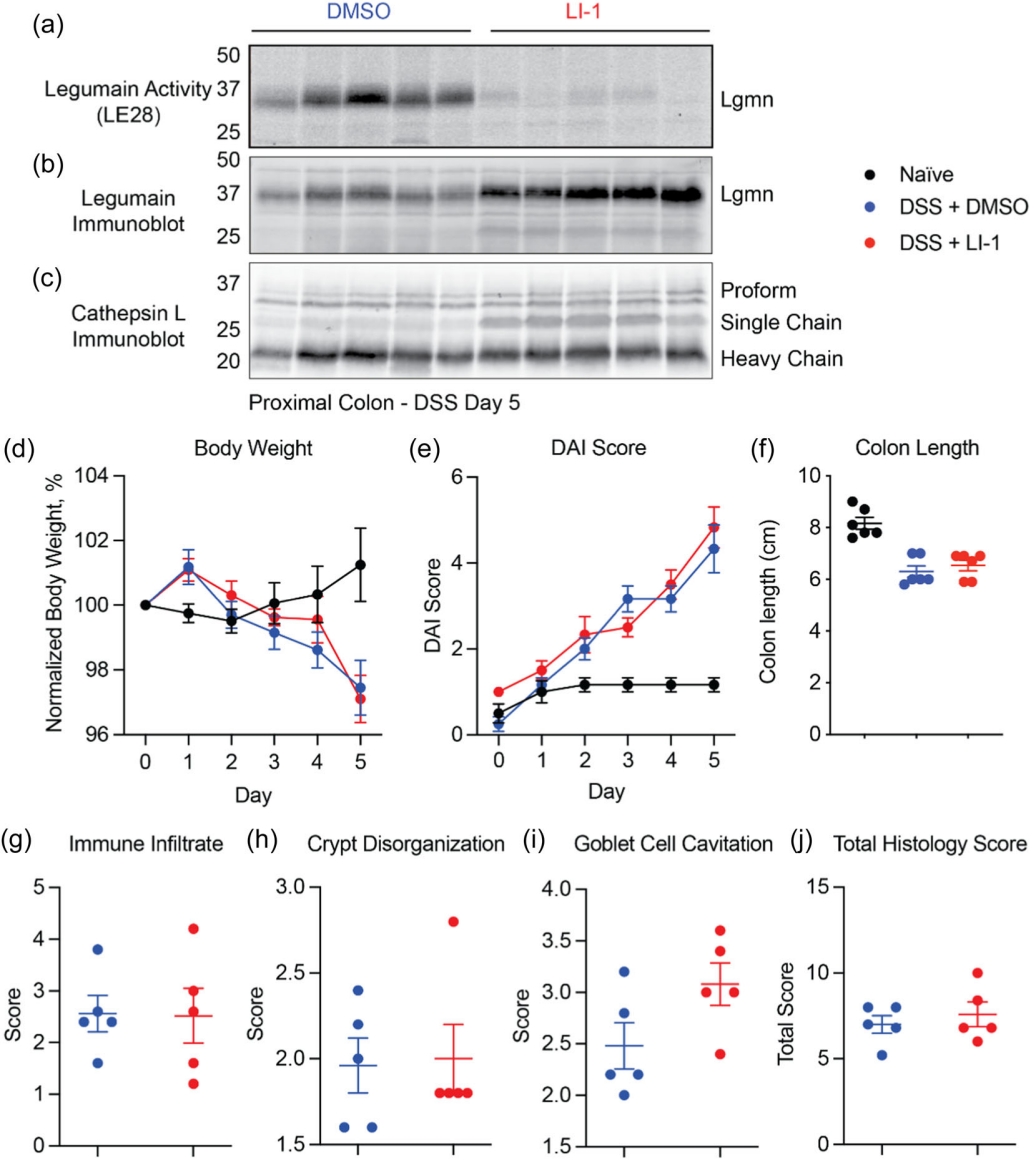
Legumain inhibition does not prevent DSS‐induced acute colitis. (a) Assessment of legumain activity in DSS‐treated proximal colons after daily treatment with DMSO or LI‐1 (25 mg/kg), as shown by in‐gel fluorescence of LE28. (b, c) Immunoblot with antibodies specific to legumain or cathepsin L on the samples shown in A. Five biological replicates are shown. (d) Body weight, shown as a percentage of starting weight, over the course of six days in naïve mice or those treated with DSS and DMSO or LI‐1). (e) Disease activity index (DAI) of mice over time. (f) Colon length at endpoint (Day 6). (g–j) Histological scoring of colon sections, including immune infiltration, crypt disorganization, goblet cell cavitation and total histology score. Error bars represent mean ± SEM, and 5–6 mice/group are reported. No significant differences between DMSO‐ and LI‐1‐treated mice were observed.

Having demonstrated that legumain activity is upregulated in models of acute colitis, we next queried whether pharmacological blockade of its activity would alter the course of disease progression. Mice were treated daily with the covalent legumain inhibitor LI‐1, which is an analogue of SD‐134 that is acetylated instead of Cbz‐capped (Lee & Bogyo, 2012) or DMSO vehicle during colitis induction with DSS. To verify the efficacy of the inhibitor, proximal colons were harvested and labeled ex vivo with the LE28 ABP. Legumain activity was almost completely abolished after LI‐1 treatment and was substantially reduced compared to DMSO‐treated mice (Figure 3a). In line with previous studies (Anderson et al., 2020; Edgington‐Mitchell et al., 2016), LI‐1 treatment led to increased total legumain levels (Figure 3b). We also observed accumulation of single‐chain cathepsin L (Figure 3c). As processing of single‐chain cathepsin to its two‐chain form is dependent on legumain activity (Anderson et al., 2020; Edgington‐Mitchell et al., 2016; Maehr et al., 2005), this provides further confirmation of LI‐1 target engagement.

Over the course of the 5‐day DSS treatment, we observed no significant differences in weight loss, DAI, or colon shortening at endpoint between DMSO‐ and LI‐1‐treated mice (Figure 3d–f). Histological evaluation of colon damage revealed no differences in immune infiltration, crypt disorganization, or goblet cell cavitation, and total damage scores were also indistinguishable (Figure 3g–j, S3).

**Figure 4:**
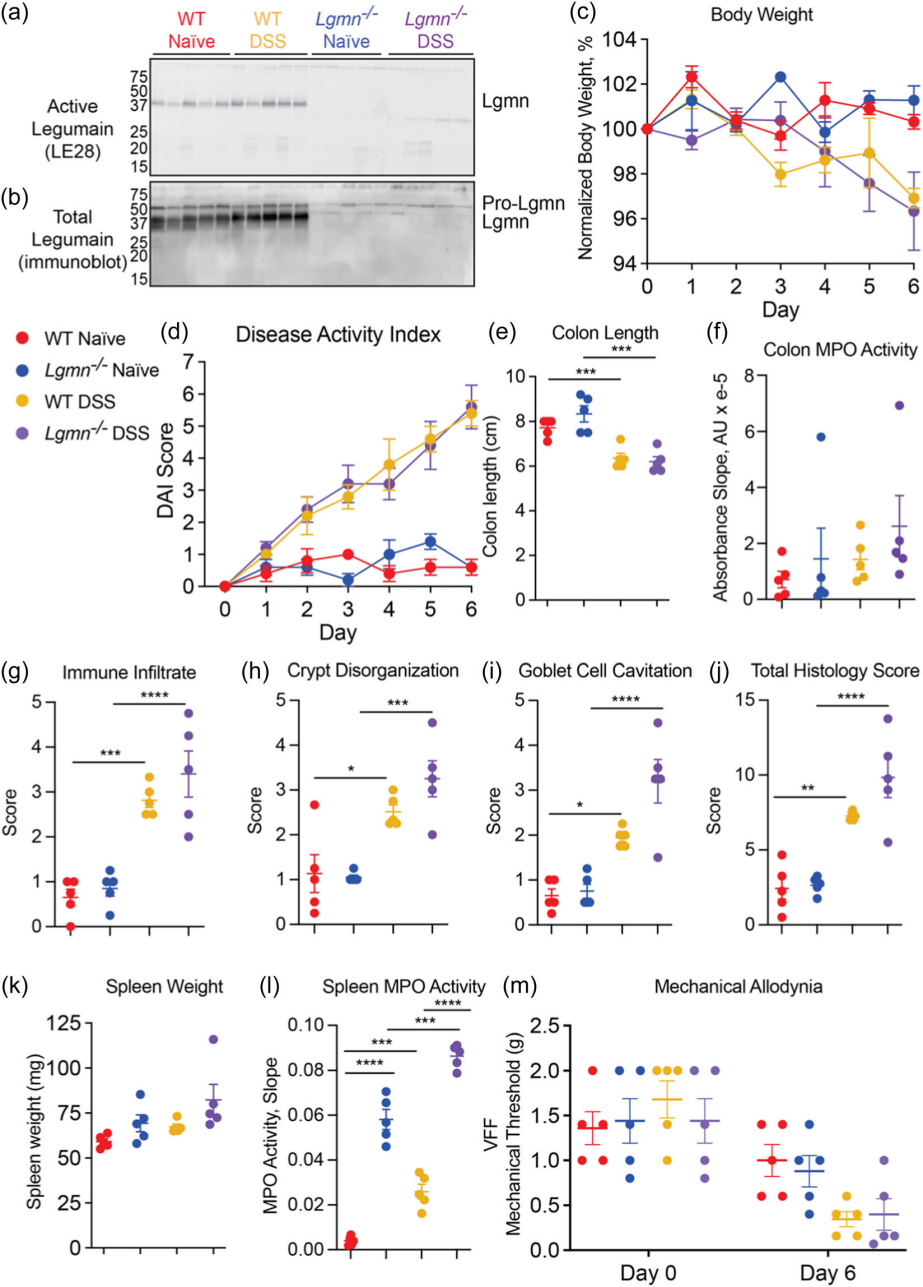
Legumain‐deficient mice are not protected from DSS‐induced colitis. (a) Wild‐type (WT) and legumain knockout (*Lgmn* ^−/−^) colon tissues from naïve or DSS‐treated mice (6 days) were lysed and labeled with LE28 ex vivo to assess legumain activity by in‐gel fluorescence. (b) Immunoblot of the samples in A with a legumain‐specific antibody. Five biological replicates are shown. (c) Body weight, shown as a percentage of starting weight, over the course of 6 days in WT and *Lgmn* ^−/−^, naïve or DSS‐treated. (d) Disease activity index (DAI) of mice over time. (e, f) Colon length and myeloperoxidase activity at endpoint (Day 6). (g–j) Histological scoring of colon sections, including immune infiltration, crypt disorganization, goblet cell cavitation and total histology score. (k–l) Spleen weight and myeloperoxidase activity at end point. Note that MPO data from naïve spleens were previously published in (Ziegler et al., 2024) but these tissues were analyzed alongside the DSS spleens. (m) Mechanical allodynia of mice at endpoint, as measured by von Frey assay of the abdomen and reported as withdrawal threshold (g). Error bars represent mean ± SEM, and five mice/group are reported. Unless indicated, significant differences between WT and *Lgmn* ^−/−^ naïve or DSS‐treated mice were not observed. ****p < 0.0001, ***p < 0.001, **p < 0.01, *p < 0.05 determined using a one‐way ANOVA with Tukey's multiple comparisons test.

Since we observed increased legumain expression in response to LI‐1 treatment, there may have been residual legumain activity over the course of colitis induction. We therefore independently examined legumain‐deficient mice in the DSS model. We confirmed loss of legumain activity in these mice using LE28 and immunoblot (Figure 4a,b). Processing of cathepsin L to its two‐chain form was lost in *Lgmn* ^−/−^ colons, providing secondary confirmation that legumain activity was lost in these tissues (Figure S4). Wild‐type and *Lgmn* ^−/−^ mice were indistinguishable in almost all parameters examined, including weight loss, DAI, colon shortening, colon MPO activity, and histological evaluation (Figure 4c–j, S5).

The weights of spleens from both naïve and DSS‐treated *Lgmn* ^−/−^ mice trended towards an increase compared to WT, but this was not significant (Figure 4k **)**. We previously reported that MPO activity was significantly elevated in naïve *Lgmn* ^*‐/‐*^ spleen (30.6‐fold compared to WT, *p* < 0.0001) (Ziegler et al., 2024). For both genotypes, splenic MPO activity was increased upon DSS treatment, and again *Lgmn* ^−/−^ exhibited significantly higher levels (3.5‐fold compared to WT, *p* < 0.0001; Figure 4l). Legumain‐deficient mice have been previously reported to exhibit splenomegaly with increased neutrophil numbers in both peripheral blood and spleen (Chan et al., 2009), and this was in line with increased abundance of neutrophil‐associated proteins measured by quantitative proteomics (Ziegler et al., 2024). From our colon MPO data (and the below proteomics analysis), there appears to be tissue‐specific differences in neutrophil load upon loss of legumain.

Given the role of PAR_2_ in promoting visceral hypersensitivity, we also examined whether legumain deficiency would alter nocifensive behaviors in this model. We observed no differences in mechanical allodynia of the abdominal region in the absence of legumain, as measured by von Frey assay (Figure 4m). Mice were also analyzed using a behavioral spectrometer to measure spontaneous behaviors in an unbiased manner (Figure S6). Overall, the naïve legumain‐deficient mice exhibited less overall activity at baseline, but this was not dependent on colitis induction, suggesting that legumain does not significantly contribute to behaviors associated with colitis.

Collectively, these results demonstrate that legumain is dispensable for the initiation of symptoms associated with DSS‐induced acute colitis and does not significantly contribute to visceral hypersensitivity.

### 3.5 Legumain‐dependent proteolysis in the naïve and inflamed gut

**Figure 5:**
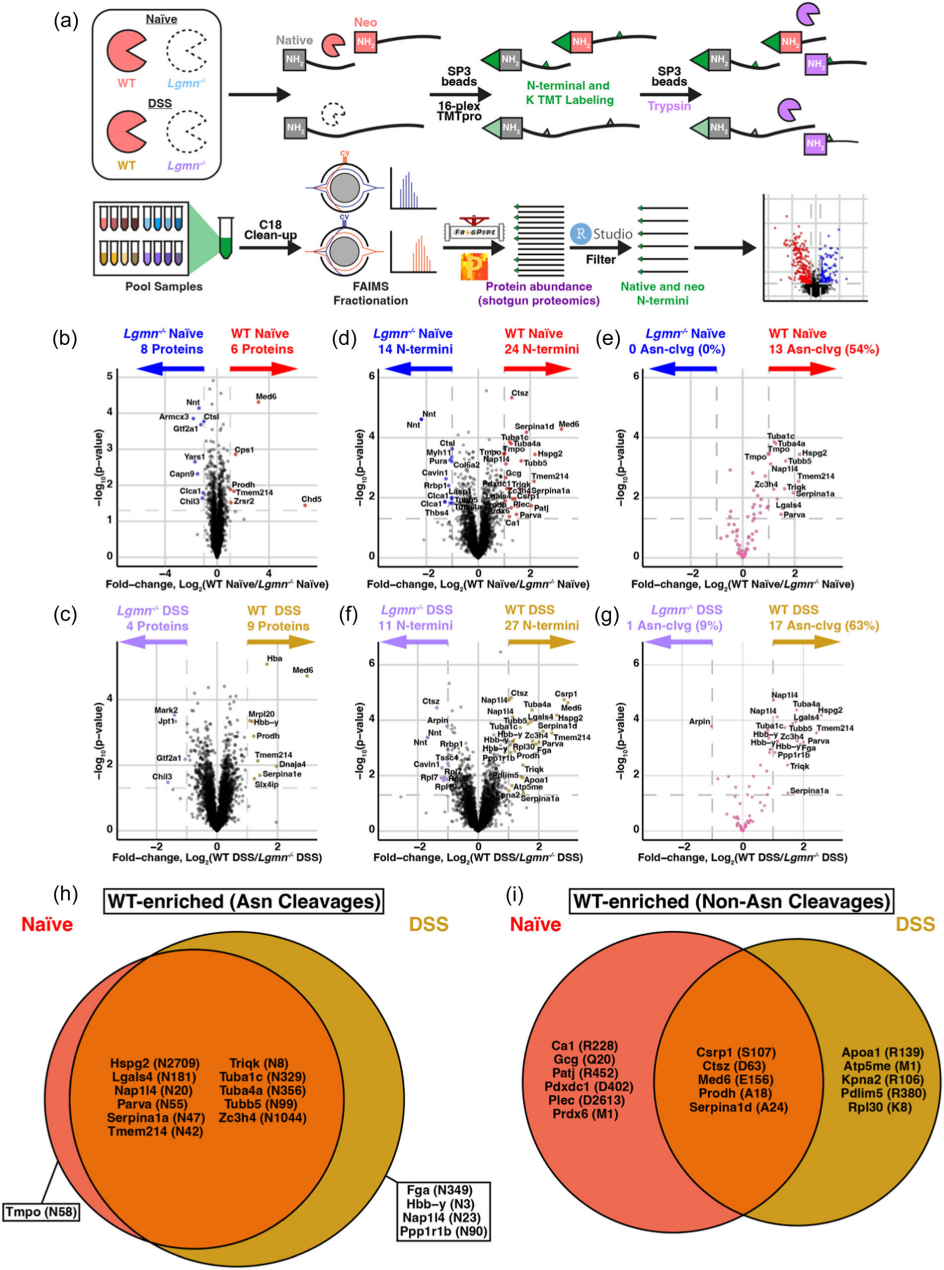
Protein abundance and proteolysis are altered in the gut in the absence of legumain. (a) Experimental workflow. Colon tissues from naïve or DSS‐treated WT or *Lgmn* ^−/−^ mice were harvested on Day 6 and analyzed by FAIMS‐facilitated N‐terminomics (*n* = 4 mice per group). Native and neo‐N‐termini were labeled with TMTpro and peptides digested by trypsin. Online gas‐phase fractionation was achieved using high‐field asymmetric wavefield ion mobility spectrometry (FAIMS) over a range of compensation voltages (CV, −20, −30, −40, −50, −60 or −70) before mass spectrometry analysis. Data were analyzed by MSFragger (Fragpipe) and Perseus. Native cleavage sites were bioinformatically enriched by filtering for N‐terminal TMTpro labeling using RStudio. Numbers shown refer to peptide‐spectrum matches present in at least three of four biological replicates in a minimum of one group. Proteins identified in naive (b) or DSS‐treated (c) colon lysates were subject to a two‐sample t test and visualized by volcano plot. Log_2_(WT/*Lgmn* ^−/−^) > |1| and −log_10_(p‐value) >1.3 were considered to be significantly enriched. (d, f) Total peptide‐spectrum matches were bioinformatically filtered for N‐terminal TMTpro labeling, indicating endogenous N‐termini. A two‐sample t test was performed, and N‐termini were visualized by volcano plot. (e, g) N‐termini were further filtered to identify peptides arising due to cleavage after asparagine residues. Asparagine‐specific (h) or non‐asparaginyl (i) cleavage events, denoted by P1 residue of cleavage site, that were significantly enriched in WT colon compared to *Lgmn* ^−/−^, detected uniquely in naive or DSS‐treated tissue or common to both.

Although we did not observe overt phenotypic differences in legumain‐deficient mice during acute colitis, we queried whether we could obtain information about its potential roles in healthy and inflamed gut and examine broad changes in proteolysis that are associated with colitis. We therefore adapted our recently developed FAIMS‐facilitated N‐terminomics pipeline to identify protease cleavage events that occur in WT and *Lgmn* ^*‐/‐*^ colon tissue, either naïve or inflamed (Ziegler et al., 2024). We applied 16‐plex TMTpro labeling to facilitate simultaneous quantification of N‐termini and total protein abundance across 16 samples (four mice per group; Figure 5a).

In this data set, we identified 19,264 unique peptides corresponding to 3688 proteins (Tables S1–S4), including 3,334 that were TMTpro labeled at the N terminus (native and protease‐generated N‐termini; Table S5–S8; Figure S7). In naïve colons, we identified six proteins that were increased in WT compared to *Lgmn* ^−/−^ tissue, and eight proteins that were reduced in abundance (Figure 5b). In DSS‐treated samples, nine proteins were increased in WT compared to *Lgmn* ^−/−^ and four were reduced (Figure 5c). Among these, five proteins were identified in both naïve and DSS‐treated samples: Prodh, Med6, TMEM214 (increased in WT); Chil3, Gtf2a1 (increased in *Lgmn* ^−/−^).

We detected 24 TMTpro‐labeled N‐terminal peptides that were enriched in WT naïve colons, which indicate cleavage events directly or indirectly mediated by legumain (Figure 5d; Table S5). Among these peptides, 13 (54%) corresponded to cleavage after asparagine residues, indicating that they may be direct substrates of legumain (Figure 5e). In DSS‐treated tissues, 27 TMTpro‐labeled peptides were enriched in WT compared to *Lgmn* ^−/−^, including 16 (62%) arising from cleavage after asparagine (Figure 5f–g; Table S6). Only one asparaginyl cleavage (4%) was detected in legumain‐deficient samples, affirming that the asparaginyl endopeptidase signature was lost in the absence of legumain (Figure 5e,g). This is also reflected in density plots, where cleavages with P1 asparagine were significantly skewed towards WT samples (Figure S8A,B). P1 arginine, however, was not enriched in either genotype.

The majority (11) of asparaginyl cleavages were detected in both naïve and DSS samples, as well as five of the nonasparagine cleavages (Figure 5h,i). Three cleavages exhibiting P1 aspartic acid were detected (Plec, Ctsz, and Pdxdc1), which may reflect the ability of legumain to cleave after this residue at acidic pH (Edgington et al., 2013).

We used pLogo (O'Shea et al., 2013) to analyze the consensus sequences for both asparagine and non‐asparagine cleavages (Figure S9A,B). Among the asparagine cleavages, the consensus sequence looked similar between naïve and DSS‐treated proteins, with methionine and serine enriched in the P2 position, glycine and alanine at P3, and aspartic acid and isoleucine at P1'. Among the non‐asparagine cleavages, which may be cleavages indirectly mediated by legumain, the motifs differed between naïve and DSS samples, suggesting unique legumain‐influenced proteases may be responsible for these differential events.

Fibrinogen alpha (Fga) was among the asparaginyl cleavages detected in DSS tissue of WT mice. In a previous analysis of proteolytic events in human wound fluids (Sabino et al., 2018), several cleavages after asparagine residues were detected in fibrinogen subunits. We therefore aimed to validate whether legumain could directly cleave fibrinogen. Indeed, when co‐incubated in acidic buffer, recombinant legumain cleaved Fg alpha, beta, and gamma in a time‐dependent manner (Figure S10). We did not observe limited proteolysis but rather loss of fibrinogen over time, suggesting that legumain may function to promote fibrinogen degradation.

We also observed a number of cleavage events that were enriched in *Lgmn* ^−/−^ tissue, including 14 in naïve and 11 in DSS, with Nnt detected in both groups (Figure 5d,f). This suggests alterations in proteolysis in the absence of legumain, potentially as a compensatory response to loss of legumain.

### 3.6 Global alterations in gut proteolysis during colitis

**Figure 6:**
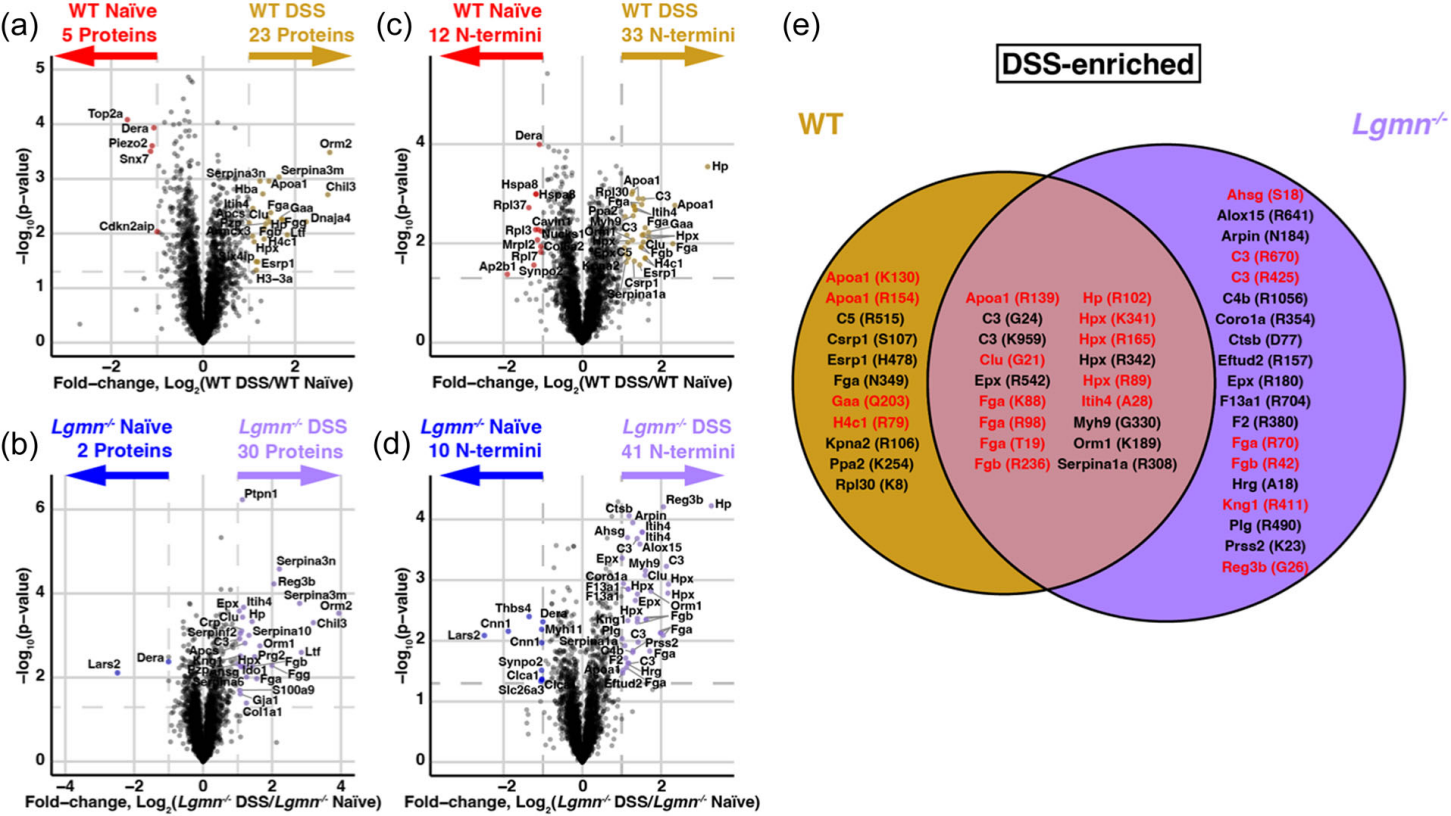
Protein abundance and proteolysis are globally altered during experimental colitis. Data from FAIMS‐facilitated N‐terminomics analysis were reanalyzed to identify proteins enriched in naïve or DSS‐treated colons of either WT or *Lgmn* ^−/−^ genotype. Identified peptides were required to be present in at least three of four biological replicates in at least one group to be considered for analysis (*n* = 4/group). Proteins identified in WT (a) or *Lgmn* ^−/−^ (b) colon lysates were subject to a two‐sample t test and visualized by volcano plot. Log_2_(DSS/Naïve) > |1| and −log_10_(p‐value) > 1.3 was considered to be significantly enriched. (c, d) Total peptide‐spectrum matches were bioinformatically filtered for N‐terminal TMT labeling, indicating endogenous N‐termini. A two‐sample *t*‐test was performed, and N‐termini were visualized by volcano plot. (e) Cleavage events in proteins, denoted by P1 residue of cleavage site, that were increased in the colon after DSS treatment, detected uniquely in WT or *Lgmn* ^−/−^ tissue or common to both. Proteins shown in red are also increased in total abundance.

Within our proteomics data set, we also examined changes in total protein abundance and proteolysis that occur after the onset of colitis (Tables S3,S4, S7,S8). In WT tissues, 23 proteins were increased in abundance in DSS samples compared to naïve, and 5 were decreased (Figure 6a). In *Lgmn* ^−/−^ samples, 30 proteins were increased in DSS tissues and 2 were decreased (Figure 6b). Among these, 15 proteins were identified as altered between naïve and DSS in both genotypes.

Among cleavage sites detected in WT tissue, 33 were enriched in DSS samples and 12 were enriched in naïve (Figure 6c). In *Lgmn* ^−/−^ tissue, 41 cleavage sites were enriched in DSS and 10 in naïve (Figure 6d). STRING‐db (v.12.0) analysis of the DSS‐enriched N‐termini (WT or *Lgmn* ^−/−^) further revealed increased cleavage of proteins related to coagulation cascades, complement activation, and platelet degranulation, irrespective of genotype (Figure S11A,B). Many of the identified cleavage sites were also identified as increased at the total protein level after DSS treatment (Figure 6e, red).

Eighteen cleavage sites were identified as enriched in both genotypes, but we also identified 11 that were unique to WT colons, and a further 19 unique to *Lgmn* ^−/−^ tissue (Figure 6e). Of the cleavages sites that were more prevalent after DSS treatment, there was no evidence of an asparaginyl endopeptidase signature, with the majority of cleavages occurring after P1 lysine or arginine residues (Figure S8C,D, S9C,D). This suggests that despite increased legumain activity during colitis, there may not be substantial change in the breadth of direct substrates. It may instead function to regulate the activity of other proteases, which could account for differences in cleavage sites identified upon DSS treatment in WT and *Lgmn* ^−/−^ tissue.

## 4 DISCUSSION

Using the activity‐based probe, LE28, we demonstrated that the cysteine protease legumain is activated in murine colon during acute experimental colitis induced by DSS or TNBS. We concluded that despite its activation, legumain does not significantly contribute to symptoms associated with acute colitis, given genetic or pharmacological loss of legumain activity did not noticeably alter DSS‐induced pathogenesis.

We previously observed a similar profile in acute pancreatitis models, where legumain does not significantly contribute to symptoms associated with acute inflammation despite significantly higher levels in inflamed tissue due to macrophage infiltration. In chronic pancreatitis models, it has recently been demonstrated that macrophage‐derived legumain contributes to fibrosis associated with prolonged inflammation, and it may promote the transition to pancreatic adenocarcinoma (Ren et al., 2020). In human pancreatitis samples, we also observed an enrichment of macrophage‐derived legumain in regions of acinar‐to‐ductal metaplasia, suggesting it may be involved in the reprogramming events that lead to cancer (Edgington‐Mitchell et al., 2016). Given the high levels of legumain associated with colorectal cancers, it is likely to play similar cancer‐promoting roles in the landscape of chronic gut inflammation (Haugen et al., 2013; Haugen et al., 2015). Chronic intestinal inflammation is associated with increased risk of developing colorectal cancers due to complex interplay between the immune cells, microbiota, and epithelial cells and activation of carcinogenic signaling pathways (Zhang et al., 2023). Patients with cancers resulting from chronic inflammation exhibit worse prognoses than those with sporadic cancers. It will therefore be paramount in the future to extend our studies to more chronic colitis and colorectal cancer models.

Given our prior observation that legumain can provoke PAR_2_‐dependent mechanical allodynia and hypersensitivity associated with oral cancer (Tu et al., 2021), we hypothesized that legumain would similarly provoke visceral hypersensitivity associated with colitis. Legumain‐deficient mice, however, were not protected from this symptom in DSS‐induced colitis. One explanation for this could be the high levels of trypsin‐like proteases in the intestinal environment. Legumain cleaves PAR_2_ at N^30^ ↓ R^31^, which is 6 residues to the N‐terminal side of the canonical trypsin cleavage site (R^36^ ↓ S^37^); thus, the legumain cleavage site would be removed in the presence of trypsin. We did not detect any PAR_2_ peptides in our proteomics datasets, which precludes further testing of this hypothesis. We speculate that legumain‐evoked pain may be more relevant in tissues with less trypsin activity, such as the oral cancer microenvironment.

In colon sections from both naïve and DSS‐treated mice, we observed legumain within colonic epithelial cells, in addition to CD68^+^ macrophages, using both the LE28 ABP and immunohistochemistry. Indeed, we confirmed legumain expression by colonocytes in cultured Caco‐2 cells. We hypothesized that legumain secreted from these cells may function to regulate permeability and barrier function within these cells, either through effects on PAR_2_ or other adhesion proteins. Using both CRISPR/Cas9 gene editing and legumain‐selective inhibitors, we did not observe significant changes in Caco‐2 monolayer permeability. Given legumain is secreted as a zymogen and has limited extracellular activity in normal cell culture conditions, it is perhaps unsurprising that no effects were detected. In the physiological context of a gut, especially during inflammation where tissues may be slightly acidotic, extracellular legumain may be active to cleave substrates to regulate critical functions of these cells. In our proteomics analyses, a number of epithelial‐derived proteins were dysregulated in *Lgmn* ^*‐/‐*^ colon, either differentially cleaved or altered in abundance.

Prodh is a mitochondrial proline dehydrogenase expressed in colonocytes. As the degradation products of proline include ATP and ROS, Prodh is proposed to have cancer‐promoting roles (Burke et al., 2020; Xu, Zhang, et al., 2023). Prodh levels were increased by twofold in the colon of WT mice compared to *Lgmn* ^−/−^, both naïve and inflamed, and its cleavage at A^18^ ↓ A^19^ was also increased by twofold, which may indicate removal of its mitochondrial transit peptide. As such, legumain may promote the development of colorectal cancer through promoting Prodh expression. Calcium‐activated chloride channel regulator 1 (Clca1) is a secreted zinc‐dependent metalloprotease that regulates chloride secretion and mucus production by goblet cells. Total protein and cleavage at R^695^ ↓ A^696^ were increased by 2.1‐fold and 2.4‐fold, respectively, in naïve *Lgmn* ^−/−^ tissue compared to WT. As Clca1 has been identified as a tumor suppressor (X. Li et al., 2017), loss of this protease in the presence of legumain may also promote tumorigenesis. Whether and how this occurs will be worthy of future investigation.

In our study, we identified 16 putative legumain substrates in the murine colon exhibiting a canonical asparaginyl endopeptidase signature, most of which were identified in both naïve and inflamed tissue. Among these, Tmpo, Nap1l4, Hspg2, Tubb5, Tuba4a, and Fga were previously identified in spleen. We previously validated direct cleavage of Tmpo by legumain and show herein that legumain can promote degradation of fibrinogen subunits. Alpha parvin (Parva) is a newly identified putative legumain substrate, which was found to be cleaved at N^55^ ↓ A^56^ (2.8‐ and 4‐fold enriched in WT Naïve and DSS colons, respectively, compared to *Lgmn* ^−/−^). This protein is a critical component of focal adhesions, anchoring the extracellular matrix to the actin cytoskeleton, and has been hypothesized to contribute to colorectal cancer progression (Bravou et al., 2015). The N terminus of alpha parvin is heavily phosphorylated, which regulates its matrix‐degrading and invasive functions (Pignatelli et al., 2012). Removal of phosphosites by legumain therefore stands to impact these cancer‐promoting functions. Galectin‐4 (Lgals4) is secreted from colonic epithelial cells and has likewise been shown to promote intestinal inflammation and tumor progression through stimulation of IL‐6 (Cao & Guo, 2016). We identified cleavage of galectin‐4 at N^181^ ↓ T^182^, which occurs between the two galectin domains. Further investigation is warranted on the impact of alpha‐parvin and galectin‐4 cleavage by legumain on inflammation and tumorigenesis.

In DSS‐treated compared to naïve samples, we observed an increase in total abundance and cleavage of proteases and protease inhibitors associated with complement and coagulation cascades as well as platelet activation. This was observed in both WT and *Lgmn* ^−/−^ tissues, suggesting legumain does not significantly impact these pathways. Several of the substrates that we identified in our screen also exhibited altered cleavage in mucosal biopsies from patients with colitis compared to healthy volunteers, although not necessarily at the same cleavage sites (C3, Hp, Rpl30, Epx, Apoa1, Coro1a, Fga, and Fgb–colitis; Clca1, Myh11–healthy) (Gordon et al., 2019). In the human data set, there was a significant enrichment in cleavages occurring after aspartic acid residues in both healthy and inflamed biopsy tissues. In support of this signature being mediated by caspases, cleaved caspase‐3 was detected by immunoblot in these biopsies. In our data, we only observed one significantly altered cleavage event occurring after aspartic acid (cathepsin B), which was enriched only in *Lgmn* ^−/−^ colitis tissue compared to *Lgmn* ^−/−^ naïve. The LE28 legumain probe is known to cross‐react with caspase‐3/‐7, but no detectable caspase labeling was detected in the in vivo‐labeled intestinal sections, suggesting either species‐specific differences or differences in sample handling postcollection.

In summary, we have identified that legumain is activated in the colon during experimental colitis. Contrary to our hypothesis, legumain does not contribute to the initial symptoms of colitis, including visceral hypersensitivity. We have generated a rich data set of proteins and proteolytic cleavage sites that are dependent on legumain in both naïve and inflamed colon tissue, which will be valuable for future studies aimed at dissecting the role of this elusive protease.

## AUTHOR CONTRIBUTIONS

Laura E. Edgington‐Mitchell conceived the study, planned all experiments, analyzed data, wrote the manuscript, and contributed funding. Alexander R. Ziegler, Bethany M. Anderson, and Rocco Latorre executed the experiments, collected data and analyzed data. Rachel M. McQuade assisted with data analysis. Antoine Dufour and Nichollas E. Scott contributed intellectually and edited the manuscript. Brian L. Schmidt and Nigel W. Bunnett provided funding and contributed in supervisory roles.

## CONFLICT OF INTEREST STATEMENT

N.W.B. is a founding scientist of Endosome Therapeutics Inc. Research in N.W.B.'s laboratory is supported in part by Takeda Pharmaceuticals, Inc.

## Supporting information

Supporting information.

Supporting information.

## References

[jcp31466-bib-0001] Anderson, B. M. , de Almeida, L. G. N. , Sekhon, H. , Young, D. , Dufour, A. , & Edgington‐Mitchell, L. E. (2020). N‐terminomics/TAILS profiling of macrophages after chemical inhibition of legumain. Biochemistry, 59(3), 329–340. 10.1021/acs.biochem.9b00821 31774660

[jcp31466-bib-0002] Bravou, V. , Antonacopoulou, A. , Papanikolaou, S. , Nikou, S. , Lilis, I. , Giannopoulou, E. , & Kalofonos, H. P. (2015). Focal adhesion proteins α‐ and β‐parvin are overexpressed in human colorectal cancer and correlate with tumor progression. Cancer Investigation, 33(8), 387–397. 10.3109/07357907.2015.1047508 26115385

[jcp31466-bib-0003] Burke, L. , Guterman, I. , Palacios Gallego, R. , Britton, R. G. , Burschowsky, D. , Tufarelli, C. , & Rufini, A. (2020). The Janus‐like role of proline metabolism in cancer. Cell Death Discovery, 6, 104. 10.1038/s41420-020-00341-8 33083024 PMC7560826

[jcp31466-bib-0004] Cao, Z. Q. , & Guo, X. L. (2016). The role of galectin‐4 in physiology and diseases. Protein & Cell, 7(5), 314–324. 10.1007/s13238-016-0262-9 27017379 PMC4853315

[jcp31466-bib-0005] Cenac, N. , Andrews, C. N. , Holzhausen, M. , Chapman, K. , Cottrell, G. , Andrade‐Gordon, P. , Steinhoff, M. , Barbara, G. , Beck, P. , Bunnett, N. W. , Sharkey, K. A. , Ferraz, J. G. P. , Shaffer, E. , & Vergnolle, N. (2007). Role for protease activity in visceral pain in irritable bowel syndrome. Journal of Clinical Investigation, 117(3), 636–647. 10.1172/JCI29255 17304351 PMC1794118

[jcp31466-bib-0006] Chan, C. B. , Abe, M. , Hashimoto, N. , Hao, C. , Williams, I. R. , Liu, X. , Nakao, S. , Yamamoto, A. , Zheng, C. , Henter, J. I. , Meeths, M. , Nordenskjold, M. , Li, S. Y. , Hara‐Nishimura, I. , Asano, M. , & Ye, K. (2009). Mice lacking asparaginyl endopeptidase developdisorders resembling hemophagocytic syndrome. Proceedings of the National Academy of Sciences, 106, 468–473. 10.1073/pnas.0809824105 PMC2626726 19106291

[jcp31466-bib-0007] Cui, Y. , Li, Q. , Li, H. , Wang, Y. , Wang, H. , Chen, W. , Zhang, S. , Cao, J. , & Liu, T. (2017). Asparaginyl endopeptidase improves the resistance of microtubule‐targeting drugs in gastric cancer through IQGAP1 modulating the EGFR/JNK/ERK signaling pathway. OncoTargets and Therapy, 10, 627–643. 10.2147/OTT.S125579 28223821 PMC5304996

[jcp31466-bib-0008] Cui, Y. , Wang, Y. , Li, H. , Li, Q. , Yu, Y. , Xu, X. , Xu, B. , & Liu, T. (2016). Asparaginyl endopeptidase promotes the invasion and metastasis of gastric cancer through modulating epithelial‐to‐mesenchymal transition. Oncotarget, 7, 34356–34370. 27102302 10.18632/oncotarget.8879 PMC5085161

[jcp31466-bib-0009] Demir, F. , Niedermaier, S. , Kizhakkedathu, J. N. , & Huesgen, P. F. (2017). Profiling of protein N‐termini and their modifications in complex samples. Methods in Molecular Biology, 1574, 35–50. 10.1007/978-1-4939-6850-3_4 28315242

[jcp31466-bib-0010] Edgington, L. E. , Verdoes, M. , Ortega, A. , Withana, N. P. , Lee, J. , Syed, S. , Bachmann, M. H. , Blum, G. , & Bogyo, M. (2013). Functional imaging of legumain in cancer using a new quenched activity‐based probe. Journal of the American Chemical Society, 135(1), 174–182. 10.1021/ja307083b 23215039 PMC4429797

[jcp31466-bib-0011] Edgington‐Mitchell, L. E. , Wartmann, T. , Fleming, A. K. , Gocheva, V. , van der Linden, W. A. , Withana, N. P. , Verdoes, M. , Aurelio, L. , Edgington‐Mitchell, D. , Lieu, T. , Parker, B. S. , Graham, B. , Reinheckel, T. , Furness, J. B. , Joyce, J. A. , Storz, P. , Halangk, W. , Bogyo, M. , & Bunnett, N. W. (2016). Legumain is activated in macrophages during pancreatitis. American Journal of Physiology‐Gastrointestinal and Liver Physiology, 311(3), G548–G560. 10.1152/ajpgi.00047.2016 27514475 PMC5075999

[jcp31466-bib-0012] Erben, U. , Loddenkemper, C. , Doerfel, K. , Spieckermann, S. , Haller, D. , Heimesaat, M. M. , Zeitz, M. , Siegmund, B. , & Kühl, A. A. (2014). A guide to histomorphological evaluation of intestinal inflammation in mouse models. International journal of clinical and experimental pathology, 7(8), 4557–4576. 25197329 PMC4152019

[jcp31466-bib-0013] Fortelny, N. , Yang, S. , Pavlidis, P. , Lange, P. F. , & Overall, C. M. (2015). Proteome TopFIND 3.0 with TopFINDer and PathFINDer: Database and analysis tools for the association of protein termini to pre‐ and post‐translational events. Nucleic Acids Research, 43(Database issue), D290–D297. 10.1093/nar/gku1012 25332401 PMC4383881

[jcp31466-bib-0014] Gecse, K. , Roka, R. , Ferrier, L. , Leveque, M. , Eutamene, H. , Cartier, C. , Ait‐Belgnaoui, A. , Rosztoczy, A. , Izbeki, F. , Fioramonti, J. , Wittmann, T. , & Bueno, L. (2008). Increased faecal serine protease activity in diarrhoeic IBS patients: A colonic lumenal factor impairing colonic permeability and sensitivity. Gut, 57(5), 591–599. 10.1136/gut.2007.140210 18194983

[jcp31466-bib-0015] Gordon, M. H. , Anowai, A. , Young, D. , Das, N. , Campden, R. I. , Sekhon, H. , Myers, Z. , Mainoli, B. , Chopra, S. , Thuy‐Boun, P. S. , Kizhakkedathu, J. , Bindra, G. , Jijon, H. B. , Heitman, S. , Yates, R. , Wolan, D. W. , Edgington‐Mitchell, L. E. , MacNaughton, W. K. , & Dufour, A. (2019). N‐terminomics/TAILS profiling of proteases and their substrates in ulcerative colitis. ACS Chemical Biology, 14(11), 2471–2483. 10.1021/acschembio.9b00608 31393699

[jcp31466-bib-0016] Guo, P. , Zhu, Z. , Sun, Z. , Wang, Z. , Zheng, X. , & Xu, H. (2013). Expression of legumain correlates with prognosis and metastasis in gastric carcinoma. PLoS One, 8(9), e73090. 10.1371/journal.pone.0073090 24023813 PMC3759407

[jcp31466-bib-0017] Haugen, M. H. , Boye, K. , Nesland, J. M. , Pettersen, S. J. , Egeland, E. V. , Tamhane, T. , Brix, K. , Maelandsmo, G. M. , & Flatmark, K. (2015). High expression of the cysteine proteinase legumain in colorectal cancer ‐ implications for therapeutic targeting. European Journal of Cancer, 51(1), 9–17. 10.1016/j.ejca.2014.10.020 25466510

[jcp31466-bib-0018] Haugen, M. H. , Johansen, H. T. , Pettersen, S. J. , Solberg, R. , Brix, K. , Flatmark, K. , & Maelandsmo, G. M. (2013). Nuclear legumain activity in colorectal cancer. PLoS One, 8(1), e52980. 10.1371/journal.pone.0052980 23326369 PMC3542341

[jcp31466-bib-0019] Hughes, C. S. , Moggridge, S. , Müller, T. , Sorensen, P. H. , Morin, G. B. , & Krijgsveld, J. (2019). Single‐pot, solid‐phase‐enhanced sample preparation for proteomics experiments. Nature Protocols, 14(1), 68–85. 10.1038/s41596-018-0082-x 30464214

[jcp31466-bib-0020] Hyun, E. , Andrade‐Gordon, P. , Steinhoff, M. , & Vergnolle, N. (2008). Protease‐activated receptor‐2 activation: A major actor in intestinal inflammation. Gut, 57(9), 1222–1229. 10.1136/gut.2008.150722 18460552

[jcp31466-bib-0021] Jafari, A. , Qanie, D. , Andersen, T. L. , Zhang, Y. , Chen, L. , Postert, B. , Parsons, S. , Ditzel, N. , Khosla, S. , Johansen, H. T. , Kjærsgaard‐Andersen, P. , Delaisse, J. M. , Abdallah, B. M. , Hesselson, D. , Solberg, R. , & Kassem, M. (2017). Legumain regulates differentiation fate of human bone marrow stromal cells and is altered in postmenopausal osteoporosis. Stem Cell Reports, 8(2), 373–386. 10.1016/j.stemcr.2017.01.003 28162997 PMC5312427

[jcp31466-bib-0022] Jimenez‐Vargas, N. N. , Pattison, L. A. , Zhao, P. , Lieu, T. , Latorre, R. , Jensen, D. D. , Castro, J. , Aurelio, L. , Le, G. T. , Flynn, B. , Herenbrink, C. K. , Yeatman, H. R. , Edgington‐Mitchell, L. , Porter, C. J. H. , Halls, M. L. , Canals, M. , Veldhuis, N. A. , Poole, D. P. , McLean, P. , … Bunnett, N. W. (2018). Protease‐activated receptor‐2 in endosomes signals persistent pain of irritable bowel syndrome. Proceedings of the National Academy of Sciences, 115(31), E7438–E7447. 10.1073/pnas.1721891115 PMC6077730 30012612

[jcp31466-bib-1000] Kong, A. T. , Leprevost, F. V. , Avtonomov, D. M. , Mellacheruvu, D. , & Nesvizhskii, A. I. (2017). MSFragger: Ultrafast and comprehensive peptide identification in mass spectrometry‐based proteomics. Nature Methods, 14(5), 513–520. 10.1038/nmeth.4256 28394336 PMC5409104

[jcp31466-bib-0023] Kovalyova, Y. , Bak, D. W. , Gordon, E. M. , Fung, C. , Shuman, J. H. B. , Cover, T. L. , Amieva, M. R. , Weerapana, E. , & Hatzios, S. K. (2022). An infection‐induced oxidation site regulates legumain processing and tumor growth. Nature Chemical Biology, 18(7), 698–705. 10.1038/s41589-022-00992-x 35332331 PMC9246868

[jcp31466-bib-0024] Latorre, R. , Hegron, A. , Peach, C. J. , Teng, S. , Tonello, R. , Retamal, J. S. , Klein‐Cloud, R. , Bok, D. , Jensen, D. D. , Gottesman‐Katz, L. , Rientjes, J. , Veldhuis, N. A. , Poole, D. P. , Thomsen, A. R. B. , Schmidt, B. L. , Pothoulakis, C. H. , Rankin, C. , Xie, Y. , Koon, H. W. , & Bunnett, N. W. (2022). Mice expressing fluorescent PAR(2) reveal that endocytosis mediates colonic inflammation and pain. Proceedings of the National Academy of Sciences, 119(6), e2112059119. 10.1073/pnas.2112059119 PMC8833192 35110404

[jcp31466-bib-0025] Latorre, R. , Ramírez‐Garcia, P. D. , Hegron, A. , Grace, J. L. , Retamal, J. S. , Shenoy, P. , Tran, M. , Aurelio, L. , Flynn, B. , Poole, D. P. , Klein‐Cloud, R. , Jensen, D. D. , Davis, T. P. , Schmidt, B. L. , Quinn, J. F. , Whittaker, M. R. , Veldhuis, N. A. , & Bunnett, N. W. (2022). Sustained endosomal release of a neurokinin‐1 receptor antagonist from nanostars provides long‐lasting relief of chronic pain. Biomaterials, 285, 121536. 10.1016/j.biomaterials.2022.121536 35533442 PMC10064865

[jcp31466-bib-0026] Lee, J. , & Bogyo, M. (2012). Synthesis and evaluation of aza‐peptidyl inhibitors of the lysosomal asparaginyl endopeptidase, legumain. Bioorganic & Medicinal Chemistry Letters, 22(3), 1340–1343. 10.1016/j.bmcl.2011.12.079 22243962 PMC3272831

[jcp31466-bib-0027] Lei, K. , Kang, S. S. , Ahn, E. H. , Chen, C. , Liao, J. , Liu, X. , Li, H. , Edgington‐Mitchell, L. E. , Jin, L. , & Ye, K. (2021). C/EBPβ/AEP signaling regulates the oxidative stress in malignant cancers, stimulating the metastasis. Molecular Cancer Therapeutics, 20(9), 1640–1652. 10.1158/1535-7163.MCT-21-0019 34158346

[jcp31466-bib-0028] Li, N. , Liu, Q. , Su, Q. , Wei, C. , Lan, B. , Wang, J. , Bao, G. , Yan, F. , Yu, Y. , Peng, B. , Qiu, J. , Yan, X. , Zhang, S. , & Guo, F. (2013). Effects of legumain as a potential prognostic factor on gastric cancers. Medical Oncology, 30(3), 621. 10.1007/s12032-013-0621-9 23740003

[jcp31466-bib-0029] Li, X. , Hu, W. , Zhou, J. , Huang, Y. , Peng, J. , Yuan, Y. , Yu, J. , & Zheng, S. (2017). CLCA1 suppresses colorectal cancer aggressiveness via inhibition of the Wnt/beta‐catenin signaling pathway. Cell Communication and Signaling, 15(1), 38. 10.1186/s12964-017-0192-z 28974231 PMC5627483

[jcp31466-bib-0030] Lohman, R. J. , Cotterell, A. J. , Suen, J. , Liu, L. , Do, A. T. , Vesey, D. A. , & Fairlie, D. P. (2012). Antagonism of protease‐activated receptor 2 protects against experimental colitis. Journal of Pharmacology and Experimental Therapeutics, 340(2), 256–265. 10.1124/jpet.111.187062 22028393

[jcp31466-bib-0031] Lunde, N. N. , Holm, S. , Dahl, T. B. , Elyouncha, I. , Sporsheim, B. , Gregersen, I. , Abbas, A. , Skjelland, M. , Espevik, T. , Solberg, R. , Johansen, H. T. , & Halvorsen, B. (2017). Increased levels of legumain in plasma and plaques from patients with carotid atherosclerosis. Atherosclerosis, 257, 216–223. 10.1016/j.atherosclerosis.2016.11.026 27940038

[jcp31466-bib-0032] Lv, J. , Liu, J. , Chao, G. , & Zhang, S. (2023). PARs in the inflammation‐cancer transformation of CRC. Clinical and Translational Oncology, 25(5), 1242–1251. 10.1007/s12094-022-03052-x 36547764

[jcp31466-bib-0033] Maehr, R. , Hang, H. C. , Mintern, J. D. , Kim, Y. M. , Cuvillier, A. , Nishimura, M. , Yamada, K. , Shirahama‐Noda, K. , Hara‐Nishimura, I. , & Ploegh, H. L. (2005). Asparagine endopeptidase is not essential for class II MHC antigen presentation but is required for processing of cathepsin L in mice. The Journal of Immunology, 174(11), 7066–7074. 10.4049/jimmunol.174.11.7066 15905550

[jcp31466-bib-0034] Matthews, S. P. , Werber, I. , Deussing, J. , Peters, C. , Reinheckel, T. , & Watts, C. (2010). Distinct protease requirements for antigen presentation in vitro and in vivo. The Journal of Immunology, 184(5), 2423–2431. 10.4049/jimmunol.0901486 20164435

[jcp31466-bib-0035] McQuade, R. M. , Stojanovska, V. , Donald, E. L. , Rahman, A. A. , Campelj, D. G. , Abalo, R. , Rybalka, E. , Bornstein, J. C. , & Nurgali, K. (2017). Irinotecan‐induced gastrointestinal dysfunction is associated with enteric neuropathy, but increased numbers of cholinergic myenteric neurons. Frontiers in Physiology, 8, 391. 10.3389/fphys.2017.00391 28642718 PMC5462962

[jcp31466-bib-0036] Murthy, R. V. , Arbman, G. , Gao, J. , Roodman, G. D. , & Sun, X. F. (2005). Legumain expression in relation to clinicopathologic and biological variables in colorectal cancer. Clinical Cancer Research, 11, 2293–2299. 15788679 10.1158/1078-0432.CCR-04-1642

[jcp31466-bib-0037] O'Shea, J. P. , Chou, M. F. , Quader, S. A. , Ryan, J. K. , Church, G. M. , & Schwartz, D. (2013). pLogo: A probabilistic approach to visualizing sequence motifs. Nature Methods, 10(12), 1211–1212. 10.1038/nmeth.2646 24097270

[jcp31466-bib-0038] Peach, C. J. , Edgington‐Mitchell, L. E. , Bunnett, N. W. , & Schmidt, B. L. (2023). Protease‐activated receptors in health and disease. Physiological Reviews, 103(1), 717–785. 10.1152/physrev.00044.2021 35901239 PMC9662810

[jcp31466-bib-0039] Perez‐Riverol, Y. , Bai, J. , Bandla, C. , García‐Seisdedos, D. , Hewapathirana, S. , Kamatchinathan, S. , Kundu, D. J. , Prakash, A. , Frericks‐Zipper, A. , Eisenacher, M. , Walzer, M. , Wang, S. , Brazma, A. , & Vizcaíno, J. A. (2022). The PRIDE database resources in 2022: A hub for mass spectrometry‐based proteomics evidences. Nucleic Acids Research, 50(D1), D543–D552. 10.1093/nar/gkab1038 34723319 PMC8728295

[jcp31466-bib-0040] Pignatelli, J. , LaLonde, S. E. , LaLonde, D. P. , Clarke, D. , & Turner, C. E. (2012). Actopaxin (α‐parvin) phosphorylation is required for matrix degradation and cancer cell invasion. Journal of Biological Chemistry, 287(44), 37309–37320. 10.1074/jbc.M112.385229 22955285 PMC3481328

[jcp31466-bib-0041] Ren, Y. C. , Zhao, Q. , He, Y. , Li, B. , Wu, Z. , Dai, J. , Wen, L. , Wang, X. , & Hu, G. (2020). Legumain promotes fibrogenesis in chronic pancreatitis via activation of transforming growth factor β1. Journal of Molecular Medicine, 98(6), 863–874. 10.1007/s00109-020-01911-0 32415356

[jcp31466-bib-0042] Róka, R. , Demaude, J. , Cenac, N. , Ferrier, L. , Salvador‐cartier, C. , Garcia‐villar, R. , Fioramonti, J. , & Bueno, L. (2007). Colonic luminal proteases activate colonocyte proteinase‐activated receptor‐2 and regulate paracellular permeability in mice. Neurogastroenterology & Motility, 19(1), 57–65. 10.1111/j.1365-2982.2006.00851.x 17187589

[jcp31466-bib-0043] Sabino, F. , Egli, F. E. , Savickas, S. , Holstein, J. , Kaspar, D. , Rollmann, M. , Kizhakkedathu, J. N. , Pohlemann, T. , Smola, H. , & Auf Dem Keller, U. (2018). Comparative degradomics of porcine and human wound exudates unravels biomarker candidates for assessment of wound healing progression in trauma patients. Journal of Investigative Dermatology, 138(2), 413–422. 10.1016/j.jid.2017.08.032 28899681

[jcp31466-bib-2000] Schaab, C. , Geiger, T. , Stoehr, G. , Cox, J. , & Mann, M. (2012). Analysis of high accuracy, quantitative proteomics data in the MaxQB database. Molecular & Cellular Proteomics (MCP), 11(3), M111.014068. 10.1074/mcp.M111.014068 PMC3316731 22301388

[jcp31466-bib-0044] Schilling, O. , & Overall, C. M. (2008). Proteome‐derived, database‐searchable peptide libraries for identifying protease cleavage sites. Nature Biotechnology, 26(6), 685–694. 10.1038/nbt1408 18500335

[jcp31466-bib-0045] Tu, N. H. , Jensen, D. D. , Anderson, B. M. , Chen, E. , Jimenez‐Vargas, N. N. , Scheff, N. N. , Inoue, K. , Tran, H. D. , Dolan, J. C. , Meek, T. A. , Hollenberg, M. D. , Liu, C. Z. , Vanner, S. J. , Janal, M. N. , Bunnett, N. W. , Edgington‐Mitchell, L. E. , & Schmidt, B. L. (2021). Legumain induces oral cancer pain by biased agonism of protease‐activated receptor‐2. The Journal of neuroscience, 41(1), 193–210. 10.1523/JNEUROSCI.1211-20.2020 33172978 PMC7786216

[jcp31466-bib-3000] Tyanova, S. , Temu, T. , Sinitcyn, P. , Carlson, A. , Hein, M. Y. , Geiger, T. , Mann, M. , & Cox, J. (2016). The Perseus computational platform for comprehensive analysis of (prote)omics data. Nature Methods, 13(9), 731–740. 10.1038/nmeth.3901 27348712

[jcp31466-bib-0046] Wang, H. , Chen, B. , Lin, Y. , Zhou, Y. , & Li, X. (2020). Legumain promotes gastric cancer progression through tumor‐associated macrophages in vitro and in vivo. International Journal of Biological Sciences, 16(1), 172–180. 10.7150/ijbs.36467 31892854 PMC6930372

[jcp31466-bib-0047] Wang, Z. H. , Liu, P. , Liu, X. , Manfredsson, F. P. , Sandoval, I. M. , Yu, S. P. , Wang, J. Z. , & Ye, K. (2017). Delta‐secretase phosphorylation by SRPK2 enhances its enzymatic activity, provoking pathogenesis in alzheimer's disease. Molecular Cell, 67(5), 812–825.e815. 10.1016/j.molcel.2017.07.018 28826672 PMC5753427

[jcp31466-bib-0048] Wang, Z. H. , Xia, Y. , Liu, P. , Liu, X. , Edgington‐Mitchell, L. , Lei, K. , Ye, K. (2021). ApoE4 activates C/EBPbeta/delta‐secretase with 27‐hydroxycholesterol, driving the pathogenesis of alzheimer's disease. Progress in Neurobiology, 202, 102032. 10.1016/j.pneurobio.2021.102032 33716161 PMC8627566

[jcp31466-bib-0049] Wu, Z. , Wang, Z. H. , Liu, X. , Zhang, Z. , Gu, X. , Yu, S. P. , Keene, C. D. , Cheng, L. , & Ye, K. (2020). Traumatic brain injury triggers APP and Tau cleavage by delta‐secretase, mediating alzheimer's disease pathology. Progress in Neurobiology, 185, 101730. 10.1016/j.pneurobio.2019.101730 31778772

[jcp31466-bib-0050] Xia, Y. , Wang, Z. H. , Liu, P. , Edgington‐Mitchell, L. , Liu, X. , Wang, X. C. , & Ye, K. (2020). TrkB receptor cleavage by delta‐secretase abolishes its phosphorylation of APP, aggravating alzheimer's disease pathologies. Molecular Psychiatry, 26, 2943–2963. 10.1038/s41380-020-00863-8 32782380

[jcp31466-bib-0051] Xu, X. , Liu, M. , Peng, K. , Yu, Y. , & Liu, T. (2023). Asparaginyl endopeptidase contributes to cetuximab resistance via MEK/ERK signaling in RAS wide‐type metastatic colorectal cancer. Clinical and Translational Oncology, 25(3), 776–785. 10.1007/s12094-022-02986-6 36609651 PMC9941237

[jcp31466-bib-0052] Xu, X. , Zhang, G. , Chen, Y. , Xu, W. , Liu, Y. , Ji, G. , & Xu, H. (2023). Can proline dehydrogenase‐a key enzyme involved in proline metabolism‐be a novel target for cancer therapy? Frontiers in Oncology, 13, 1254439. 10.3389/fonc.2023.1254439 38023181 PMC10661406

[jcp31466-bib-4000] Yu, F. , Haynes, S. E. , & Nesvizhskii, A. I. (2021). IonQuant enables accurate and sensitive label‐free quantification with FDR‐controlled match‐between‐runs. Molecular & Cellular Proteomics, 20, 100077. 10.1016/j.mcpro.2021.100077 33813065 PMC8131922

[jcp31466-bib-0053] Zecha, J. , Satpathy, S. , Kanashova, T. , Avanessian, S. C. , Kane, M. H. , Clauser, K. R. , Mertins, P. , Carr, S. A. , & Kuster, B. (2019). TMT labeling for the masses: A robust and cost‐efficient, in‐solution labeling approach. Molecular & Cellular Proteomics, 18(7), 1468–1478. 10.1074/mcp.TIR119.001385 30967486 PMC6601210

[jcp31466-bib-0054] Zhang, H. , Shi, Y. , Lin, C. , He, C. , Wang, S. , Li, Q. , Sun, Y. , & Li, M. (2023). Overcoming cancer risk in inflammatory bowel disease: New insights into preventive strategies and pathogenesis mechanisms including interactions of immune cells, cancer signaling pathways, and gut microbiota. Frontiers in immunology, 14, 1338918. 10.3389/fimmu.2023.1338918 38288125 PMC10822953

[jcp31466-bib-0055] Ziegler, A. R. , Dufour, A. , Scott, N. E. , & Edgington‐Mitchell, L. E. (2024). Ion mobility‐based enrichment‐free N‐terminomics analysis reveals novel legumain substrates in murine spleen. Molecular & Cellular Proteomics, 23(2), 100714. 10.1016/j.mcpro.2024.100714 38199506 PMC10862022

