# Supplementary material for: N‐terminomics profiling of naïve and inflamed murine colon reveals proteolytic signatures of legumain
Source: J Cell Physiol. 2024 Oct 11;240(1):e31466. doi: 10.1002/jcp.31466 (PMC11735880; doi:10.1002/jcp.31466)
Supplement: Supplementary file 1 — Supporting information. [file JCP-240-0-s002.pdf]

Supplementary Information for

N-terminomics profiling of naïve and inflamed murine colon reveals proteolytic signatures of legumain

Alexander R. Ziegler[1], Bethany M. Anderson[1], Rocco Latorre[2], Rachel M. McQuade[3], Brian L. Schmidt[4], Nigel W. Bunnett[2], and Nicholas E. Scott[5], Laura E. Edgington-Mitchell[1#]

[1]Department of Biochemistry and Pharmacology, Bio21 Molecular Science and Biotechnology Institute, The University of Melbourne, Parkville, Victoria, 3052 Australia.

[2]Department of Molecular Pathobiology, New York University College of Dentistry, New York, New York, 10010 USA.

[3]Department of Anatomy and Physiology, The University of Melbourne, Parkville, Victoria, 3052 Australia.

[4]Department of Oral and Maxillofacial Surgery, New York University College of Dentistry, Bluestone Center for Clinical Research, New York, New York, 10010 USA.

[5]Department of Microbiology and Immunology, Peter Doherty Institute, The University of Melbourne, Parkville, Victoria, 3052 Australia.

Contents

Table S1 Protein changes in naïve colon of WT and *Lgmn*$^{-/-}$ mice
Table S2 Protein changes in DSS colon of WT and *Lgmn*$^{-/-}$ mice
Table S3 Protein changes in WT colon under naïve and DSS conditions
Table S4 Protein changes in *Lgmn*$^{-/-}$ colon under naïve and DSS conditions
Table S5 N-termini changes in naïve colon of WT and *Lgmn*$^{-/-}$ mice
Table S6 N-termini changes in DSS colon of WT and *Lgmn*$^{-/-}$ mice
Table S7 N-termini changes in WT colon under naïve and DSS conditions
Table S8 N-termini changes in *Lgmn*$^{-/-}$ colon under naïve and DSS conditions
Supplementary Methods
Table S9 Legumain knockout primers for CRISPR
Figure S1 Specificity of legumain antibody
Figure S2 Impact of legumain activity on epithelial permeability
Figure S3 Impact of legumain inhibition on colon histology
Figure S4 Impact of legumain deletion of cathepsin L processing in the colon
Figure S5 Impact of legumain deletion on colon histology
Figure S6 Impact of legumain deletion on spontaneous locomotion
Figure S7 Summary of proteins and peptides identified by FAIMS-facilitated N-terminomics
Figure S8 Density plots of cleavage sites identified in mouse colon
Figure S9 Consensus motifs of cleavage sites identified in mouse colon
Figure S10 Legumain cleaves fibrinogen in vitro
Figure S11 Reactome pathways associated with DSS-enriched cleavage events

Supplementary Methods

CRISPR/Cas9 deletion of *Lgmn*

Legumain was knocked out in Caco-2 cells using the CRISPR/Cas9 strategy previously outlined (Ran et al., 2013). Two different primers were designed to target different regions of the human legumain gene using CHOPCHOP (Labun et al., 2019) (**Table S9**). Forward and reverse primers were annealed into an oligo-duplex by incubating 1 µL of each (100 µM) in 6 µL nuclease-free (NF water) with 1 µL 10x T4 ligase buffer (1x final, New England Biolabs, B0201S) and 1 µL T4 PNK enzyme (New England Biolabs, M0201L) for 30 min at 37 °C, followed by enzyme inactivation at 95 °C for 5 min. The samples were held at 94 °C for an additional 20 s prior to decreasing the temperature to 25 °C at a rate of 1 °C per min and holding for 20 s. This was cycled for a total of 70 times. The annealed oligos (1 µL) were ligated into the pSpCas9 (BB)-2A-GFP vector pre-digested with *BsbI* (10 ng/µL) in 5 µL NF-water with 1 µL 10x T4 ligase buffer (1x final) and 1 µL T4 DNA ligase (New England Biolabs, M0202L) for 60 min at room temperature. The plasmid was transformed into DH5α-competent *E.coli* by incubating on ice for 10 min and subsequent heat shock at 42 °C for one min before returning to ice for a further two min. Bacteria were suspended in Luria Broth (LB), incubated with shaking for one hour at 37 °C and plated on LB-agar plates containing ampicillin (100 µg/mL) for an overnight incubation. The next day, individual colonies were grown in LB for one hour at 37 °C and the presence of legumain single-guide RNA was checked by colony PCR. Successfully transformed bacteria were further grown overnight and plasmid DNA was extracted using the Qiagen miniprep kit according to manufacturer's instructions. Plasmids were sent to the Australian Genome Research Facility (AGRF) for sequencing.

For cell transfection, Caco-2 cells were seeded in a 6-well plate (7.0×10^5 per well) and left to adhere overnight. Plasmids were diluted in 100 µL OptiMEM (Gibco, 31985062) with 6 µL X-tremeGENE 9 Transfection Reagent (Roche, XTG9-RO) and left at room temperature for 10 min prior to adding over cells in a dropwise manner. A control was included which had no addition of plasmid. Following 24 h of transfection, the cells were collected using 0.25% trypsin-EDTA, pelleted by centrifugation (300 *x g*, 5 minutes) and resuspended in 300 µL 5% FBS (v/v) in PBS. Cell suspensions were passed through 100 µm filter and sent to the Murdoch's Children Research Institute (MCRI) for single-cell sorting based on GFP fluorescence (nozzle and pressure, 100 µm at 22 psi). Individual clones were grown as above and tested for legumain activity and expression using LE28 for live labelling and immunoblot as described.

Table S9: Legumain (LGMN) knockout primers for CRISPR.

Gene	Forward primer (5'-3')	Reverse primer (5'-3')
Human legumain sgRNA-1 (targets exon 8)	CACCGATAACATCAA TGGTAGGTGG	AAACCCTACCATTGA TGTTATCCGC
Human legumain sgRNA-2 (targets exon 3)	CACCGATCACAACGA TCTGTTCGTC	AAACGACGAACAGAT CGTTGTGATC

Caco-2 permeability assay

Wild-type (WT) and legumain-deficient (*LGMN*$^{-/-}$) Caco-2 cells were seeded (2.0×10^5) in triplicate into 0.4 µm pore polyester membrane Transwell inserts (StemCell Technologies). Media was changed after 16 h to prevent the formation of multiple layers. Cells were allowed to grow for 21 days with media changed every second day. Where indicated, SD-134 was added (10 µM) with DMSO used as a vehicle control and incubated at 37 °C with 5% CO_2 overnight. SD-134 is an analogue of LI-1 containing a Cbz cap in place of acetylation; they both target legumain with similar specificity but SD-134 is used here due to its relative ease of synthesis. Media was then removed and replaced with Hank's Balanced Salt Solution (HBSS, Sigma) containing HEPES (25 mM, pH 7.4, Sigma) to equilibrate the inserts for 30 min at 37 °C. The apical compartment was then replaced with HBSS/HEPES with fluorescein isothiocyanate (FITC)-dextran (3-5 kDa, 100 µg/mL, Sigma) and cell permeability was quantified by taking 200 µL from the basolateral compartment every 20 min for 2 h and then 4 h. FITC fluorescence was measured in the ClarioStar Omega plate reader (BMG Technologies) with excitation at 490 nm and emission at 520 nm and the raw data visualised.

Cleavage assay

Direct cleavage of fibrinogen by legumain was assessed *in vitro* using recombinant proteins. Recombinant activated human legumain (gift from Hans Brandstetter, 1.5 µg/µL stock, 0.015 µg/µL final concentration) was incubated with recombinant fibrinogen (Sigma F3879, 1 µg/µL stock, 0.1 µg/µL final concentration) for 3 h at 37°C in acetate buffer (50 mM sodium acetate (ChemSupply), 100 mM sodium chloride (EMSURE), pH 5.5). Fibrinogen was also incubated without legumain as a negative control. The reaction was quenched with addition of 5x sample buffer (1x final concentration) and cleavage was analysed on a 15% SDS-PAGE gel poured in-house. The gel was stained with 0.1% Coomassie brilliant blue G-250 dye (Biorad) in 50% methanol, 10% acetic acid for 30 min at room temperature prior to destaining in 30% ethanol,

10% methanol for 10 min three times. Gels were rinsed in MilliQ water overnight prior to imaging on the Typhoon 5 IRlong channel.

Supplementary References

Labun, K., Montague, T. G., Krause, M., Torres Cleuren, Y. N., Tjeldnes, H., & Valen, E. (2019). CHOPCHOP v3: expanding the CRISPR web toolbox beyond genome editing. *Nucleic Acids Res, 47*(W1), W171-W174. doi:10.1093/nar/gkz365

Ran, F. A., Hsu, P. D., Wright, J., Agarwala, V., Scott, D. A., & Zhang, F. (2013). Genome engineering using the CRISPR-Cas9 system. *Nat Protoc, 8*(11), 2281-2308. doi:10.1038/nprot.2013.143

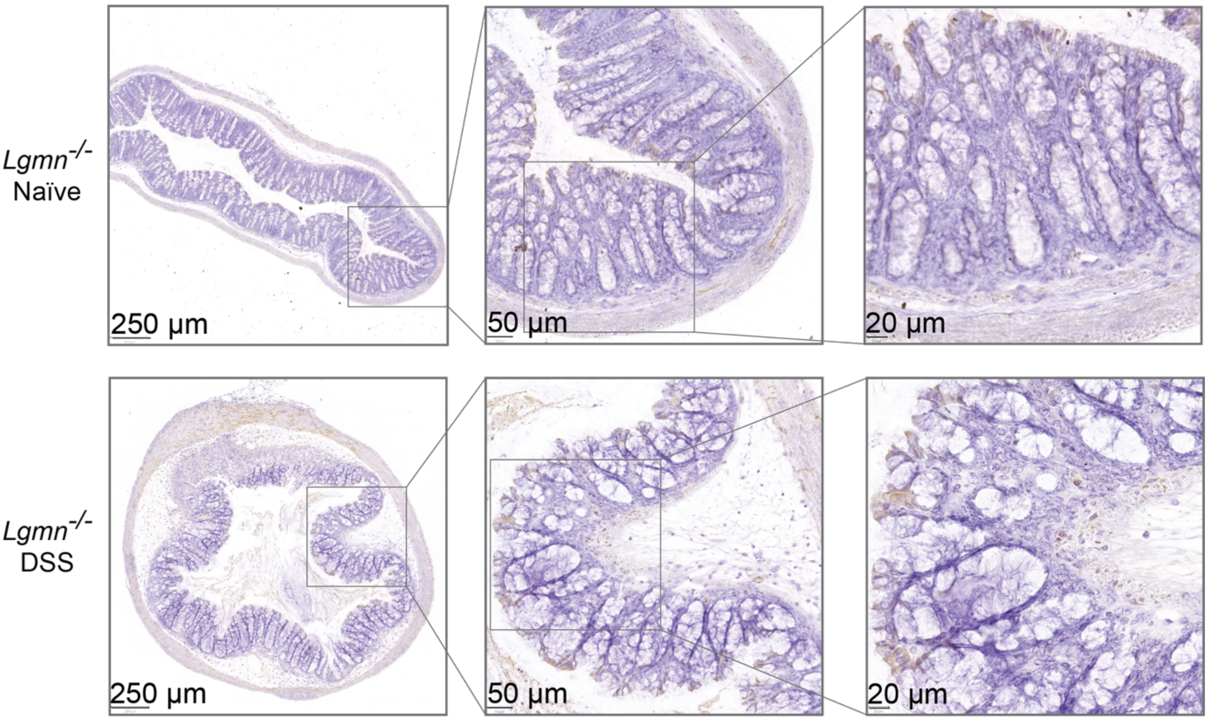

Figure S1. Specificity of anti-legumain antibody. Anti-legumain immunoreactivity in colon tissue sections from naïve or DSS-treated *Lgmn*$^{-/-}$ mice. Sections were stained alongside wild-type tissues shown in Figure 2. Note this antibody was also validated by immunoblot in *Lgmn*$^{-/-}$ colon tissue (see Figure 4).

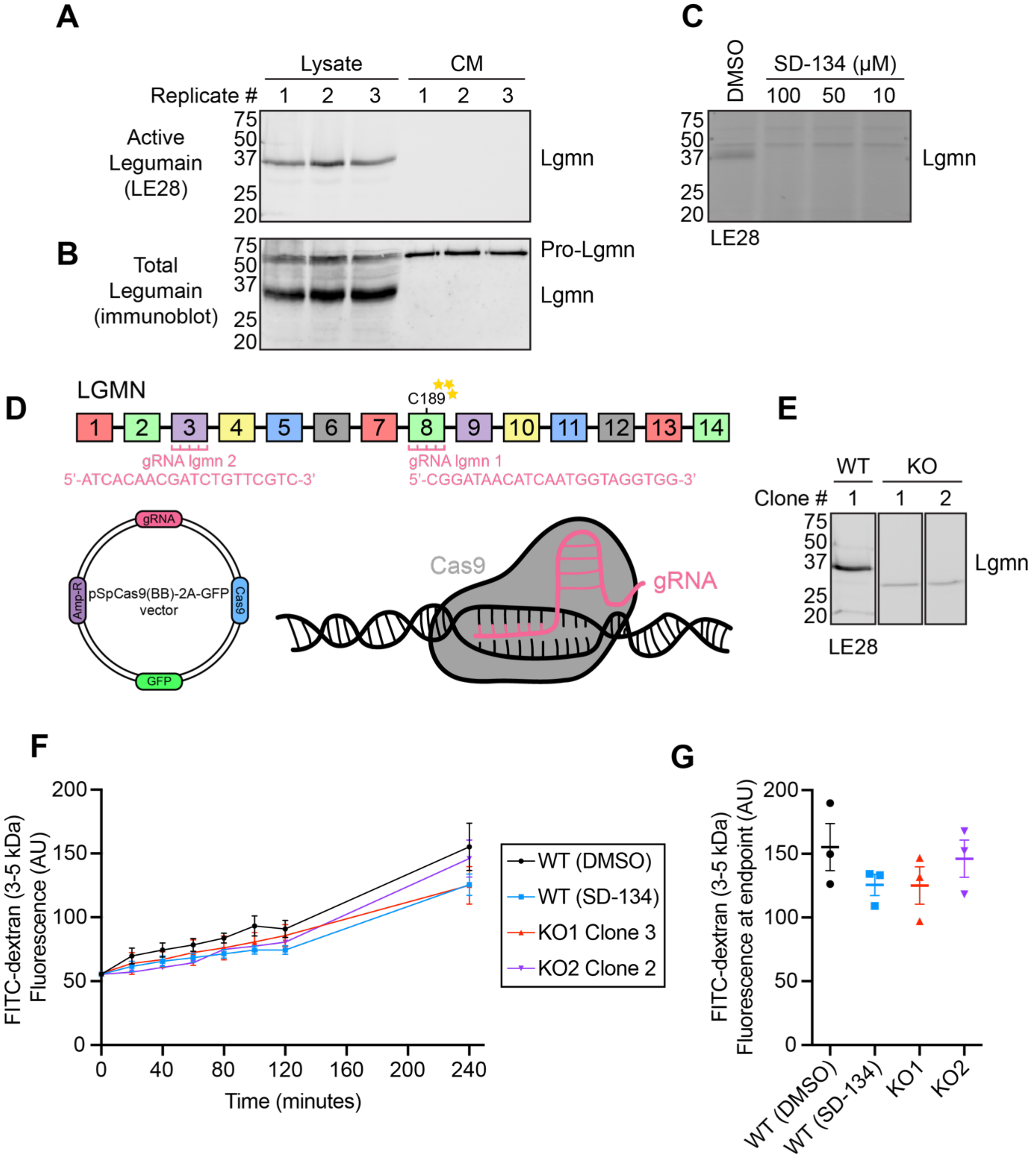

Figure S2. Impact of legumain activity on epithelial permeability. Intracellular and secreted) **(A)** legumain activity as shown by in-gel fluorescence of live-labeled LE28 and **(B)** total legumain levels shown by immunoblot in Caco-2 cells (CM = conditioned media). Three technical replicates are shown. **(C)** Effect of legumain inhibitor SD-134 (10, 50, 100 μM) on legumain activity in Caco-2 cells, as shown by in-gel fluorescence of lysate-labeled LE28. **(D)** Schematic of CRISPR/Cas9 gene-editing strategy to knock out *Lgmn* in Caco-2 cells. (E) Legumain activity measured by in-gel fluorescence of live-labeled LE28 in a single-cell WT clone and 2 single-cell *LGMN*$^{-/-}$ clones. FITC-dextran (3-5 kDa) flux from Caco-2 monolayers over time **(F)** and at end point **(G).** Three technical replicates for each group are shown, and this was repeated in at least three separate experiments. Error bars represent means ± SEM. No significant differences in permeability were detected upon loss of legumain activity.

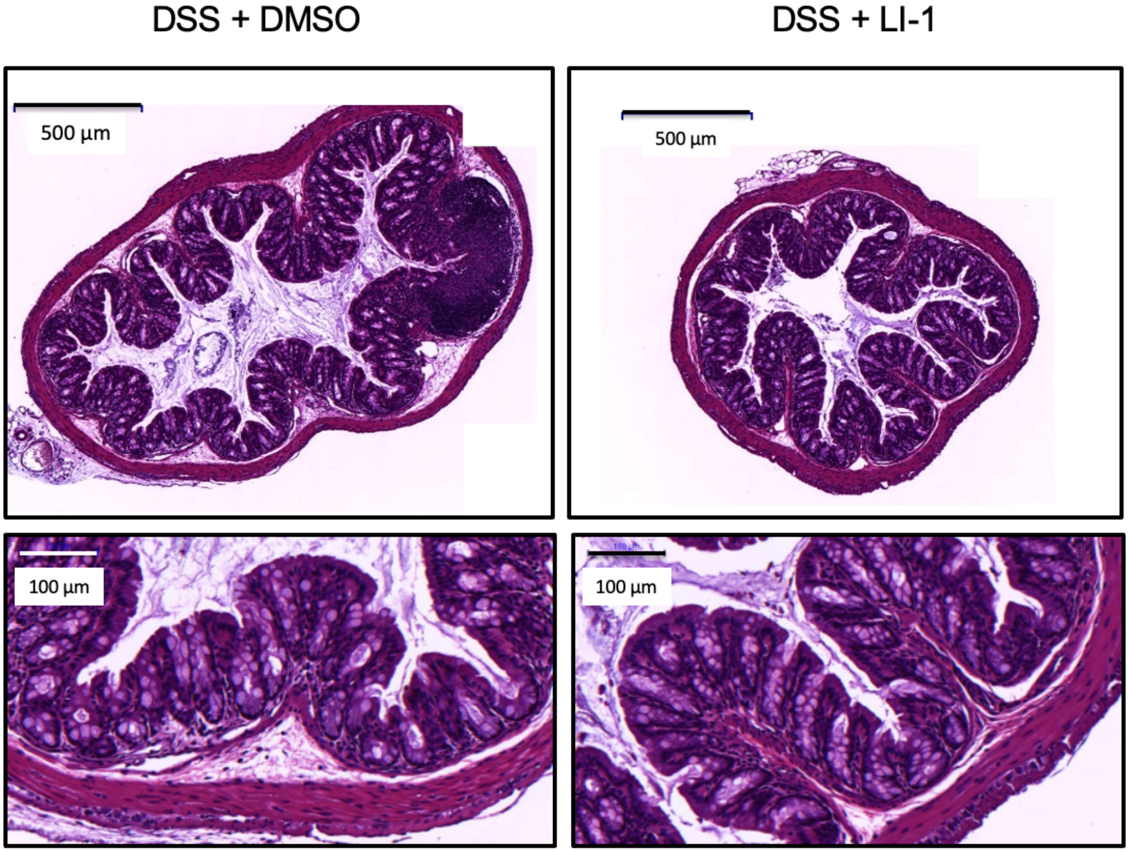

Figure S3. Impact of legumain inhibition on colon histology. Representative images of H&E-stained colon sections obtained from naïve or DSS-treated mice, corresponding to scoring shown in Figure 3G-J.

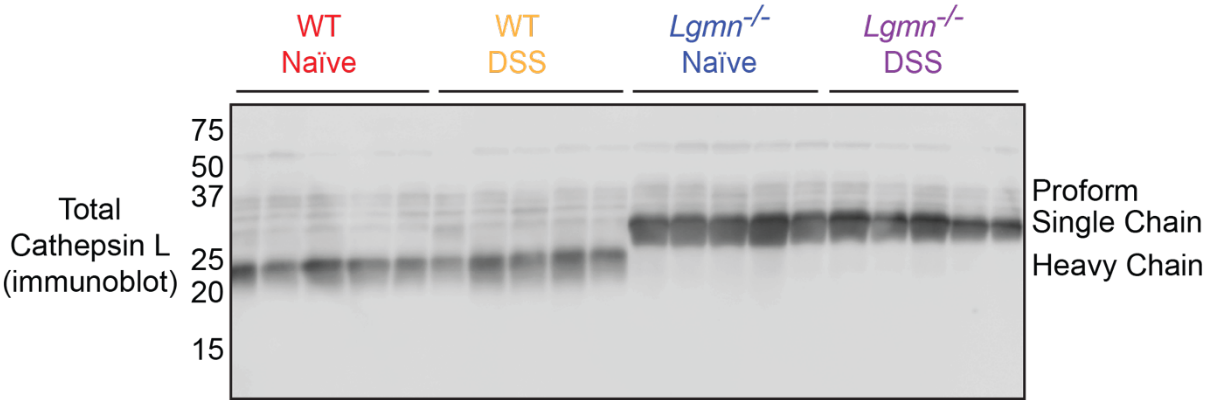

Figure S4. Impact of legumain deletion on cathepsin L processing in the colon. WT or *Lgmn*$^{-/-}$ colons from naïve or DSS-treated mice were immunoblotted with a cathepsin L-specific antibody.

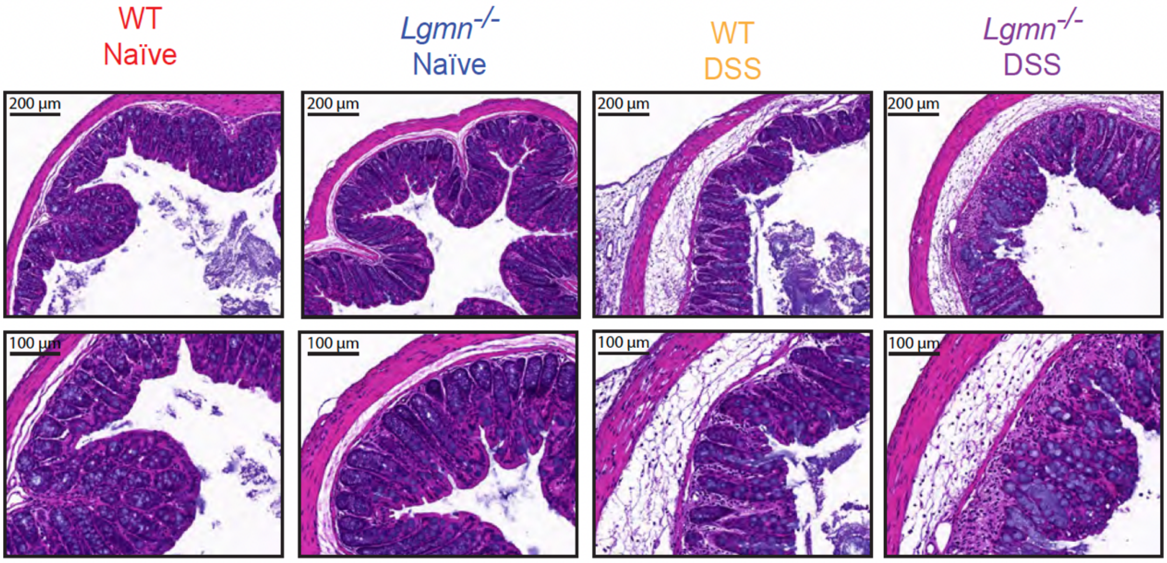

Figure S5. Impact of legumain deletion on colon histology. Representative images of H&E-stained colon sections obtained from naïve or DSS-treated WT and *Lgmn*$^{-/-}$ mice, corresponding to scoring shown in Figure 4G-J.

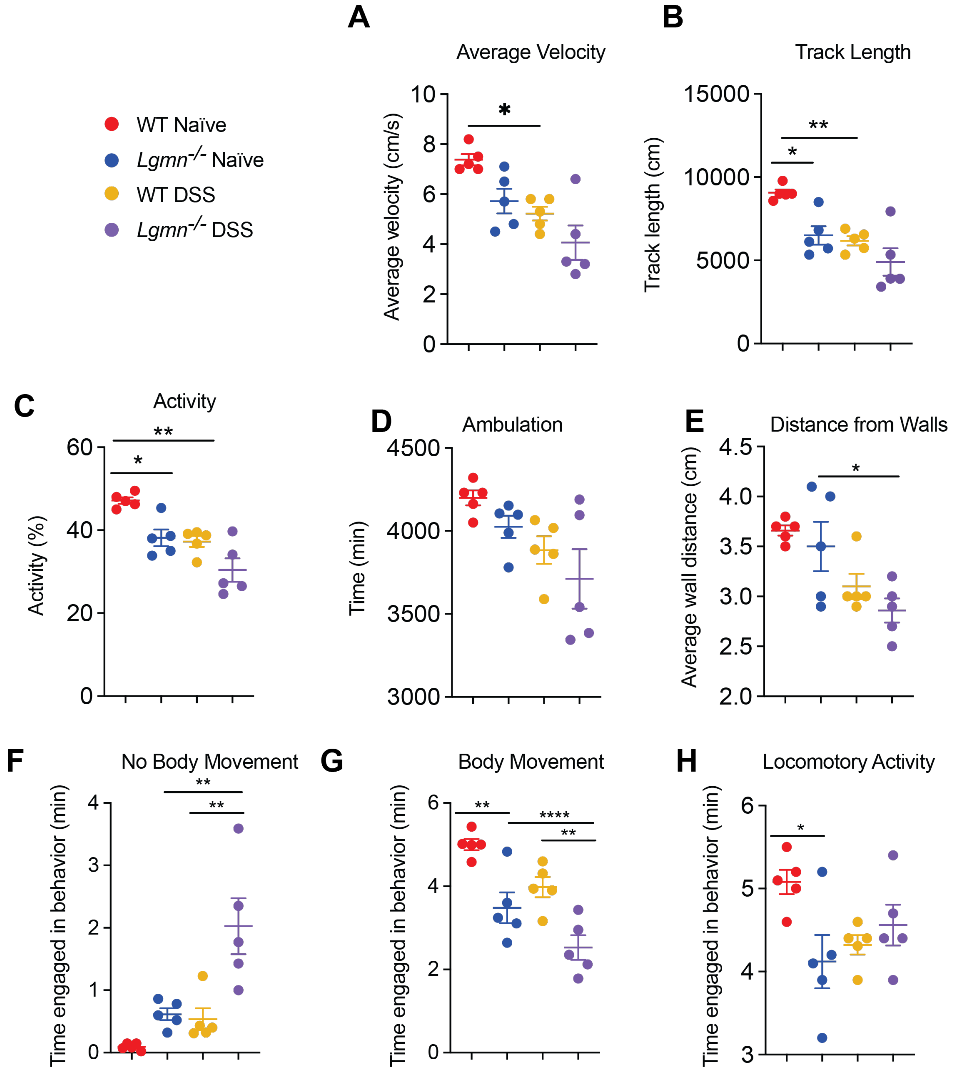

Figure S6. Impact of legumain deletion on spontaneous locomotion. Naïve or DSS-treated WT or *Lgmn*$^{-/-}$ mice were assessed for spontaneous activity using a behavioral spectrometer on day 6. **(A)** Average velocity (speed in cm/s) measured in the whole arena. **(B)** Total length of the track length (cm) measured in the whole arena. **(C)** Activity, reported as percentage of time in which the animal's movement speed exceeded the activity threshold. **(D)** Ambulation behavior defined as a spontaneous short-term acceleration (parameters analyzed: slow, medium, and fast movement). **(E)** Distance of the animal's body point to the nearest wall, averaged over all video frames. **(F)** Time spent sitting still. **(G)** Time spent in movement. **(H)** Time engaged in locomotory activity. Error bars represent means ± SEM and 5 mice/group are reported. Data were analyzed by one-way ANOVA with Tukey's multiple comparisons test, and $p<0.05$ was considered significant. * $p<0.05$; ** $p<0.01$, **** $p<0.0001$

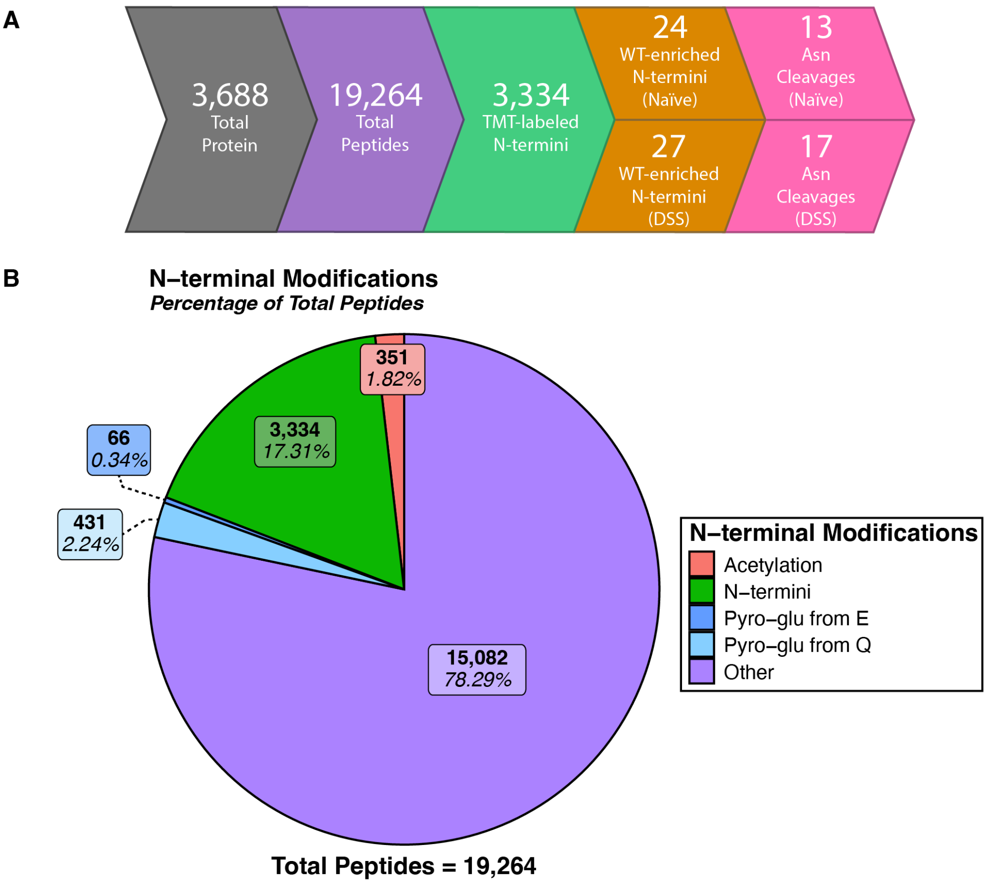

Figure S7. Summary of proteins and peptides identified by FAIMS-facilitated N-terminomics. **(A)** Summary of proteins and peptides identified in FAIMS-fractionated naïve and DSS-treated mouse colon lysates. Total peptide-spectrum matches were bioinformatically filtered for N-terminal TMT labeling indicating endogenous N-termini. N-termini were further filtered for those arising due to cleavage after asparagine residues. **(B)** Summary of N-terminal modifications identified shown as a percentage of all peptides.

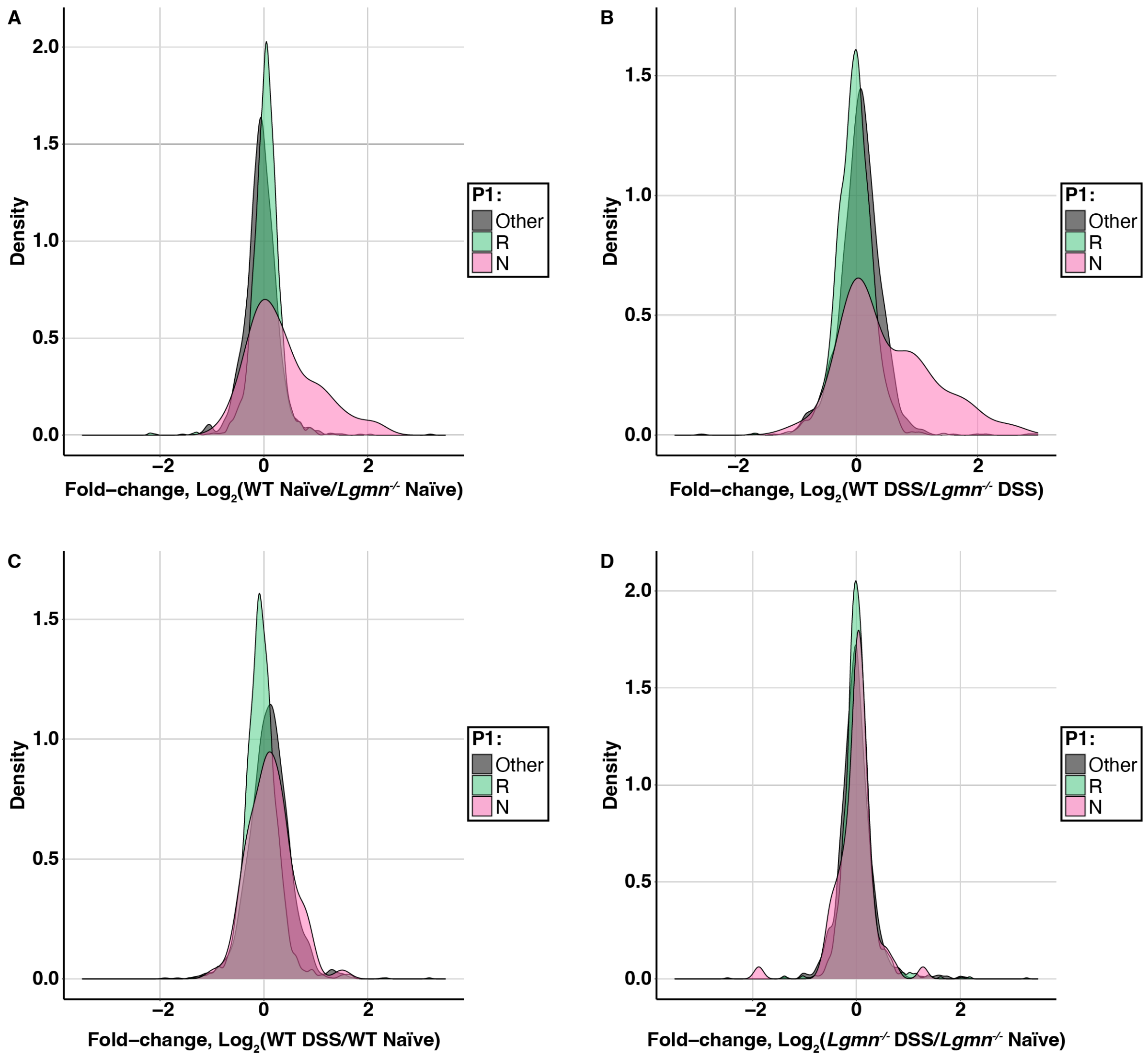

Figure S8. Density plots of cleavage sites identified in mouse colon. TMT-labeled peptides identified in colons from naïve or DSS-treated WT or *Lgmn*$^{-/-}$ mice were filtered according to whether they arose from cleavage after asparagine (N; pink), arginine (R; green) or other residues (grey). The indicated fold change was plotted against density. **(A)** Log_2(WT Naïve/*Lgmn*$^{-/-}$ Naïve). **(B)** Log_2(WT DSS/*Lgmn*$^{-/-}$ DSS). **(C)** Log_2(WT DSS/WT Naïve). **(D)** Log_2(*Lgmn*$^{-/-}$ DSS/*Lgmn*$^{-/-}$ Naïve).

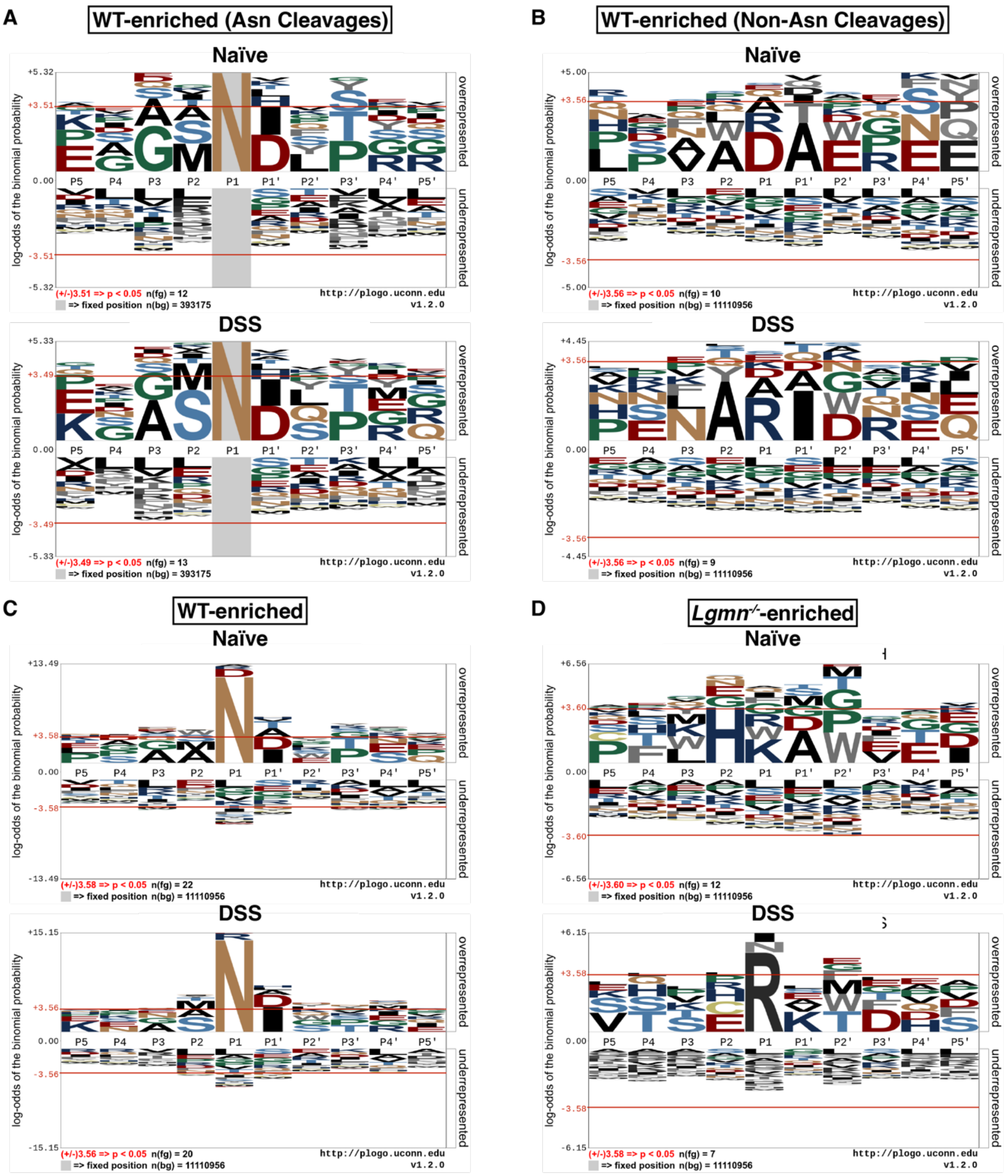

Figure S9. Consensus motifs of cleavage sites identified in mouse colon**.** Sequence motifs of N-termini significantly enriched in WT naïve or DSS-treated colons arising from cleavage after P1 asparagine **(A)** or other residues **(B)**. Motifs enriched in naïve or DSS treated colons from WT **(C)** or Lgmn-/- **(D)** colons. Created using plogo (O'Shea et al. 2013). Overrepresented amino acids appear above and underrepresented below the x-axis ($p < 0.05$).

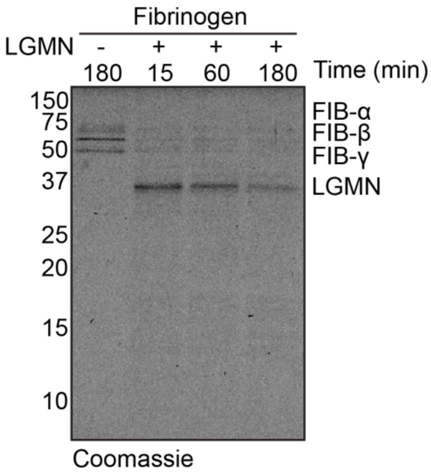

Figure S10. Legumain cleaves fibrinogen in vitro. Recombinant fibrinogen was incubated in the absence and presence of legumain for the indicated time. Proteins were resolved by SDS-PAGE and the gel was stained with Coomassie to visualize cleavage products.

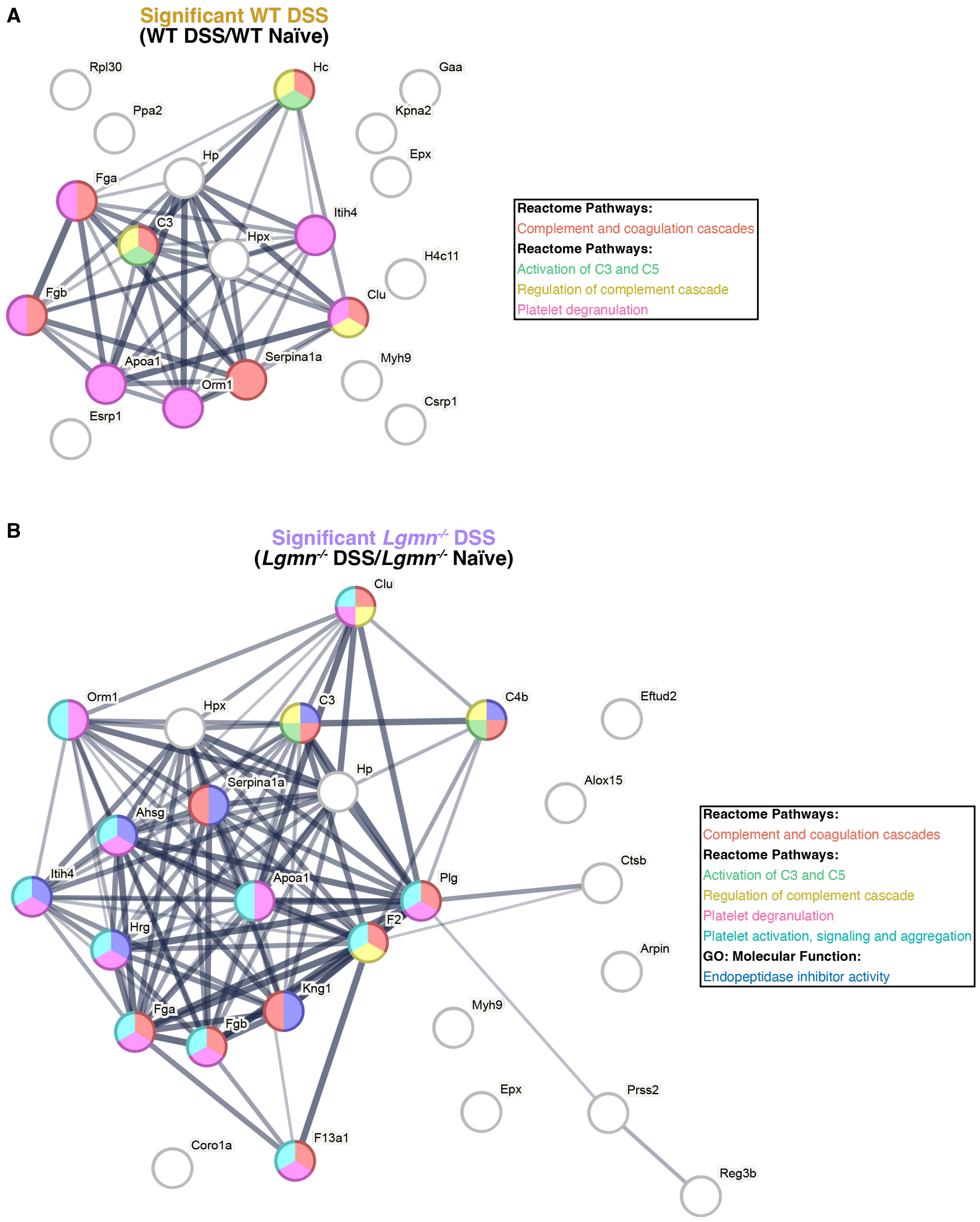

Figure S11. Reactome pathways associated with DSS-enriched cleavage events. STRING-db (v.12.0) analysis of the DSS-enriched N-termini from WT **(A)** or *Lgmn*$^{-/-}$ **(B)** mice (confidence = 0.400, false discovery rate = 5%). Line thickness corresponds to the confidence of interaction. Colors are indicated in the legend.
